# Single-cell profiling of human dura and meningioma reveals cellular meningeal landscape and insights into meningioma immune response

**DOI:** 10.1186/s13073-022-01051-9

**Published:** 2022-05-10

**Authors:** Anthony Z. Wang, Jay A. Bowman-Kirigin, Rupen Desai, Liang-I Kang, Pujan R. Patel, Bhuvic Patel, Saad M. Khan, Diane Bender, M. Caleb Marlin, Jingxian Liu, Joshua W. Osbun, Eric C. Leuthardt, Michael R. Chicoine, Ralph G. Dacey, Gregory J. Zipfel, Albert H. Kim, David G. DeNardo, Allegra A. Petti, Gavin P. Dunn

**Affiliations:** 1 Department of Neurological Surgery, Washington University School of Medicine, St. Louis, MO USA; 2 Department of Pathology and Immunology, Washington University School of Medicine, St. Louis, MO USA; 3 Andrew M. and Jane M. Bursky Center for Human Immunology and Immunotherapy Programs, Washington University School of Medicine, St. Louis, MO USA; 4 Brain Tumor Center, Washington University School of Medicine/Siteman Cancer Center, St. Louis, USA; 5 Department of Neurosurgery, Massachusetts General Hospital, Boston, MA USA; 6 Division of Anatomic and Molecular Pathology, Department of Pathology and Immunology, Washington University School of Medicine, St. Louis, MO USA; 7 Washington University School of Medicine, St. Louis, MO USA; 8 Arthritis & Clinical Immunology Human Phenotyping Core, Oklahoma Medical Research Foundation, Oklahoma City, OK USA; 9 Department of Genetics, Washington University School of Medicine, St. Louis, MO USA; 10 McDonnell Genome Institute, Washington University School of Medicine, St. Louis, MO USA; 11 Division of Oncology-Molecular Oncology, Department of Internal Medicine, Washington University School of Medicine, St. Louis, MO USA; 12 Department of Medicine, Washington University School of Medicine, St. Louis, MO USA

**Keywords:** Single-cell RNA sequencing, Dura, Meninges, Imaging mass cytometry

## Abstract

**Background:**

Recent investigations of the meninges have highlighted the importance of the dura layer in central nervous system immune surveillance beyond a purely structural role. However, our understanding of the meninges largely stems from the use of pre-clinical models rather than human samples.

**Methods:**

Single-cell RNA sequencing of seven non-tumor-associated human dura samples and six primary meningioma tumor samples (4 matched and 2 non-matched) was performed. Cell type identities, gene expression profiles, and T cell receptor expression were analyzed. Copy number variant (CNV) analysis was performed to identify putative tumor cells and analyze intratumoral CNV heterogeneity. Immunohistochemistry and imaging mass cytometry was performed on selected samples to validate protein expression and reveal spatial localization of select protein markers.

**Results:**

In this study, we use single-cell RNA sequencing to perform the first characterization of both non-tumor-associated human dura and primary meningioma samples. First, we reveal a complex immune microenvironment in human dura that is transcriptionally distinct from that of meningioma. In addition, we characterize a functionally diverse and heterogenous landscape of non-immune cells including endothelial cells and fibroblasts. Through imaging mass cytometry, we highlight the spatial relationship among immune cell types and vasculature in non-tumor-associated dura. Utilizing T cell receptor sequencing, we show significant TCR overlap between matched dura and meningioma samples. Finally, we report copy number variant heterogeneity within our meningioma samples.

**Conclusions:**

Our comprehensive investigation of both the immune and non-immune cellular landscapes of human dura and meningioma at single-cell resolution builds upon previously published data in murine models and provides new insight into previously uncharacterized roles of human dura.

**Supplementary Information:**

The online version contains supplementary material available at 10.1186/s13073-022-01051-9.

## Background

The central nervous system (CNS) in vertebrates is encased by three layers of tissue that together comprise the meninges [1]. The outermost layer of tissue is the dura mater, the middle layer is the arachnoid mater, and the innermost layer is the pia mater, which adheres to the brain surface. The dura layer performs important structural roles in the CN S[2]. Specifically, this layer protects the underlying brain and spinal cord, harbors the large vascular sinus and lymphatic vessels through which venous and lymphatic drainage of the brain traverses, and creates intracranial compartments that divide the cerebral hemispheres and separate them from the cerebellum of the posterior fossa [2–4]. Combined, the dura and arachnoid mater form a water-tight seal to contain cerebrospinal fluid which originates from the choroid plexus and bathes the brain before exiting through arachnoid granulations, in addition to other routes such as the cribriform plate [5, 6]. Thus, the dura is critical in establishing the anatomic compartments of the brain and in performing other protective roles.

Beyond its structural roles, the meninges consist of cells which also perform critical functional roles in the CNS. The embryonic meninges influence the development of the skull, neuronal migration and anatomic positioning, neurogenesis and blood vessel development, and the establishment of basement membranes of the pia and the glia limitans [reviewed in [1, 7]]. Recently, there has been a growing appreciation that the dura also harbors vital immunologic functions, in addition to its role as a physical barrier in the innate immune response of the brain, thereby supporting the view that the meninges represent a dynamic immune microenvironment involved in organizing CNS immune responses. First, several studies in mice have shown that the dura harbors a range of immune cell types including macrophages, monocytes, dendritic cells (DCs), and T and B cells [8–10]. Second, meningeal immunity is critical to immune responses against stroke, traumatic brain injury, infection, and cancer [11–14]. Finally, recent identification of lymphatic channels in the dura has illuminated new mechanisms by which the CNS interacts with systemic immunity [15, 16]. Several examples have demonstrated that modulating various functions of the dura can alter the immune response. For example, investigators showed that the CNS anti-tumor immune response can be attenuated by ligation of cervical lymphatics originating from the dura [7] and, conversely, that CNS anti-tumor immune responses can be enhanced by the induction of dural lymphangiogenesis [17]. Furthermore, clinicians have explored endovascular embolization of the middle meningeal artery—which perfuses the dura—for the treatment of chronic subdural hematomas [18]. Thus, clarifying the cellular composition of dura may enable a better understanding of this tissue site, with important translational implications.

Because much of our understanding of meningeal biology stems almost entirely from pre-clinical models, we focused our work on characterizing meningeal composition in patients undergoing surgery for the resection of intracranial meningiomas as this is one of the few scenarios in which dura resection is clinically indicated. Meningiomas are common, typically benign, tumors originating from within the meninges and treated by surgical resection of the meningioma and nearby surrounding margin of dura, some of which is not grossly associated with the tumor as determined by the surgeon [19, 20]. Herein, we report the first characterization of human dura using single-cell RNA sequencing (scRNA-seq) by profiling the surrounding non-tumor-associated dura and a subset of matched meningiomas from patients undergoing surgical resection. In total, seven non-tumor-associated human dura samples, four matched primary meningioma tumor samples, and two non-matched primary meningioma tumor samples were analyzed. We show using scRNA-seq that human dura consists of diverse immune, endothelial, and mesenchymal cell types. We supplemented these observations with imaging mass cytometry (IMC), which allowed us to investigate the spatial relationships among these cell types, and immunohistochemistry (IHC), which allowed us to compare the expression of several markers between matched dura and tumor samples. Moreover, from the scRNA-seq data, we observed cellular heterogeneity and functional diversity within each cell population characterized. In patient-matched dura and meningioma tumors, we observed that immune cell states were distinct within each tissue. Additionally, using single-cell TCR sequencing, we show that dura that is tumor-adjacent, but not tumor-attached; harbors clonotypic T cell diversity; and shares T cell clonotypes with adjacent meningioma tumor tissue. Finally, we provide evidence of copy number heterogeneity in primary meningioma tumor samples at the single-cell level. Together, these findings provide further support that the dura is a dynamic anatomic tissue site and suggest cellular pathways by which the immune response to meningiomas evolve.

## Methods

### Experimental design

The objective of this study was to characterize the cellular composition of human dura and meningioma at a single-cell resolution. The study design involved performing 3′ and 5′ single-cell RNA sequencing, with V(D) J enrichment for select samples, in addition to imaging mass cytometry and immunohistochemistry.

### Patient recruitment and sample collection

Adult patients undergoing neurosurgical intervention at Barnes-Jewish Hospital were screened. Selection criteria included (1) age > 18 years and (2) presence of intracranial meningioma with clinical indications for surgical resection. All samples were collected from patients undergoing surgical resection for a primary meningioma tumor, except for SAMPLE06 (DURA06) which was collected from a patient undergoing surgical resection for a recurrent meningioma tumor. Prior to surgery, informed consent was obtained from patients meeting selection criteria following the Washington University School of Medicine Institutional Review Board Protocol #202107071. During surgical resection, specimens were placed in normal saline and immediately maintained on ice pending further processing. Clinical characteristics are summarized in Additional file 1: Table S1.

### Sample extraction and preparation

Dura and matched tumor samples (SAMPLE02, 05, 06, 08, 09, 10, 11, and 13) were collected from the operating room on ice. Excess sample was blotted dry and frozen in Fisher Healthcare Tissue-Plus Optimal Cutting Temperature (O.C.T.) Compound (Fisher Scientific) on dry ice and stored at –80°C. To disaggregate the remaining samples, samples were placed in a sterile-filtered medium containing 10%FBS (Lonza), IMDM (Lonza), 2 mg/mL collagenase A (Roche), and 2 mg/mL collagenase D (Roche) and macerated into small pieces with a scalpel. Samples were incubated overnight at 37°C with 5% CO_2_, with gentle pipetting intermittently to encourage further disaggregation. The following morning, after the collagen matrix had completely dissolved and the cells had dissociated into a single-cell suspension, the cell suspension was passed through a 100-μm strainer, followed by RBC lysis with ACK buffer (Lonza), followed by passing through a 70-μm strainer and then a 40-μm strainer. If a significant portion of the cells were dead, samples were subjected to EasySep^TM^ dead cell removal per manufacturer’s protocol (Stemcell Technologies). If the suspension had considerable debris, samples were subjected to debris removal solution according to manufacturer’s instructions (Miltenyi Biotec). Cells were resuspended in 10% FBS/IMDM in preparation for construction of single-cell libraries.

Meningioma samples (MEN104 and MEN108) were collected from the operating room on ice and the majority of the sample was macerated with a scalpel. Samples were processed following the human tumor dissociation kit (Miltenyi Biotec) using the tough gentleMACS program on the gentleMACs Octo Dissociator with Heaters (Miltenyi Biotec). Samples were then passed through a 70-μm filter, subjected to RBC lysis with ACK buffer (Lonza), and then sorted with CD45 MicroBeads (Miltenyi Biotec). Both CD45-positive and CD45-negative fractions were then submitted for sequencing. Excess sample was washed in PBS and fixed in 10% formalin for 24 h. Afterwards, excess samples were transferred to 70% ethanol and embedded in paraffin.

### Single-cell RNA sequencing

Cell suspensions were prepared according to the manufacturer’s protocol (10x genomics) for 3′ v3 single-cell sequencing or 5′ single-cell sequencing with TCR enrichment (Additional file 1: Table S1). For both methods, gel beads in emulsion (GEMs) were generated from a mixture of cell suspension combined with the GEM beads subjected to emulsion production by the Chromium Controller. cDNA was prepared after the GEM generation and barcoding, followed by the GEM-RT reaction and bead cleanup steps. Purified cDNA was amplified for 10–14 cycles before being cleaned up using SPRIselect beads. Samples were then run on a tape station or Bioanalyzer to determine the cDNA concentration. TCR enrichments were done on the full-length cDNA 5′ gene expression libraries (GEX). Both GEX and Enriched TCR libraries were prepared as recommended by the 10x Genomics Chromium Single Cell V(D) J Reagent Kits (v1 Chemistry) user guide with appropriate modifications to the PCR cycles based on the calculated cDNA concentration. For sample preparation on the 10x Genomics platform for 3′v3 libraries, the Chromium Single Cell 3′ GEM, Library & Gel Bead Kit v3 (PN-1000075) with the Chromium Single Cell Chip B Kit (PN-1000154) were used. For 5′ libraries the Chromium Single Cell 5′ Library and Gel Bead Kit (PN-1000006), Chromium Single Cell A Chip Kit (PN-1000152), Chromium Single Cell V(D) J Enrichment Kit, Human, T cell (96 rxns) (PN-1000005), and Chromium Single Index Kit T (PN-1000213) were used. The concentration of each library was accurately determined through qPCR utilizing the KAPA library Quantification Kit according to the manufacturer’s protocol (KAPA Biosystems/Roche) to produce cluster counts appropriate for the Illumina NovaSeq6000 instrument. Normalized libraries were sequenced on a NovaSeq6000 S4 Flow Cell using the XP workflow and a 151×10×10×151 sequencing recipe according to manufacturer protocol for 5′ sequencing and a 28×8×98 sequencing recipe according to manufacturer protocol for 3′v3 sequencing. For both sequencing approaches, a median sequencing depth of 50,000 reads/cell was targeted for each Gene Expression Library and 5000 reads/cell for each V(D) J (T cell) library generated from the 5′ sequencing library.

### Single-cell RNA-seq data processing of dura and meningioma samples

Raw sequencing data was processed with the CellRanger pipeline (10x Genomics, default settings, version 3.0.1) mapped onto a human genome GRCh38-3.0.0. All seven dura samples were then processed using the Seurat R package [21] and cells that contained fewer than 500 features, more than 10% mitochondrial transcripts, and a nCount value greater than the 93rd percentile of each individual sample were removed. Cells containing greater than 6000 nFeatures were removed from the DURA08 sample. Samples were then batched according to sequencing technology (3′ or 5′ sequencing) and each batch was individually log normalized after which variable features were selected according to default settings. Both batches were then integrated using FindIntegrationAnchors and IntegrateData. Principal component analysis was then performed and the optimal number of principal components (PCs) was determined based upon results from the elbow plots, jackstraw resampling, and PC expression heatmaps (*n*=50). Dimensionality reduction and visualization were performed with the uniform manifold approximation and projection (UMAP) algorithm [22] (Seurat implementation) and unsupervised graph-based clustering was performed at a resolution of 0.7. Cell cycle phase was assessed based on expression of phase-specific genes following methodology provided by Seurat [23].

Similarly, four paired dura and meningioma samples were processed together using the Seurat R package. Similar filtering criteria were applied, but samples were merged, rather than batched and integrated (because the same sequencing technology was used), then normalized. Variable features were selected according to default settings and principal component analysis was performed. The optimal number of principal components was determined (*n*=50) and dimensionality reduction and visualization were performed with the UMAP algorithm. Unsupervised graph-based clustering was performed at a resolution of 0.7 and cell cycle phase was similarly assessed.

Finally, DURA09, MEN09, MEN104.1 (CD45+ fraction), MEN104.2 (CD45− fraction), MEN108.1 (CD45+ fraction), and MEN108.2 (CD45− fraction) samples were processed together using the Seurat R package. Similar filtering criteria were applied, and samples were merged and normalized. Variable features were selected according to default settings and principal component analysis was performed. The optimal number of principal components was determined (*n*=40) and dimensionality reduction and visualization were performed with the UMAP algorithm. Unsupervised graph-based clustering was performed at a resolution of 0.7 and cell cycle phase was similarly assessed.

Differentially expressed genes of each cluster resolved by unsupervised graph-based clustering were determined using a Wilcoxon rank-sum test-based function. These genes, along with commonly defined markers (Additional file 1: Table S2), were used to identify cell identity.

Subpopulations of cells were isolated based on cell type classification (i.e., immune, non-immune, monocyte/macrophage, etc.), after which each subpopulation was rescaled. Variable genes were not recalculated for integrated data sets and recalculated for non-integrated data sets. Principal component analysis was performed and an appropriate number of principal components selected (dura immune cells: *n*=30, dura myeloid cells: *n*=20, dura DCs: *n*=15, dura non-immune cells: *n*=30, dura endothelial cells: *n*=20, dura fibroblasts: *n*=20, dura and tumor immune cells: *n*=30, dura and tumor myeloid cells: *n*=15, dura and tumor DCs: *n*=15, dura and tumor macrophages: *n*=15, dura and tumor non-immune cells: *n*=30, aggregated tumor cells: *n*=30, MEN09 tumor cells: *n*=20, MEN104 tumor cells: *n*=15, and MEN108 tumor cells: *n*=14). Dimensionality reduction and visualization were performed with the UMAP algorithm and unsupervised graph-based clustering was performed at the following resolutions (dura immune cells: 1.0, dura myeloid cells: 0.6, dura DCs: 0.9, dura non-immune cells: 0.9, dura endothelial cells: 0.7, dura fibroblasts: 0.8, dura and tumor immune cells: 0.7, dura and tumor myeloid cells: 0.8, dura and tumor DCs: 0.7, dura and tumor monocyte/macrophages: 0.9, dura and tumor non-immune cells: 0.8, aggregated tumor cells: 0.7, MEN09 tumor cells: 0.8, MEN104 tumor cells: 0.8, and MEN108 tumor cells: 0.7).

### Immunohistochemistry validation of antibodies and antibody conjugation for imaging mass cytometry

Purchased antibodies (Additional file 1: Table S3) were initially tested via immunohistochemistry. Respective positive control tissues for each antibody were tested (Novus Biologicals). Slides were first baked in an oven at 56 °C overnight to melt the paraffin wax, then placed in xylene for 20 min, and rehydrated in the following metal-free solutions of ethanol for 5 min each: 100%, 100%, 95%, 95%, 80%, 80%, 70%, and 70%. After rehydration, slides were placed in metal-free water for 5 min on an orbital shaker after which they were incubated in pH 9 IHC Antigen Retrieval Solution (Invitrogen) at 96°C for 30 min. Slides were cooled in the antigen retrieval solution for 10 min at room temperature, followed by a 10-min wash in metal-free water and a 10-min wash in metal-free PBS. The tissues on the slides were outlined with a hydrophobic barrier pen (Liquid Blocker) and a solution of 3% bovine serum albumin (BSA) in PBS was placed on the tissues within the hydrophobic barriers for 45 min at room temperature. The primary antibody solution was prepared at manufacturer-recommended dilutions in PBS with a final concentration of 0.5% BSA and subsequently added following removal of the 3% BSA solution. The primary antibody solution was incubated on the tissues overnight at 4°C. Slides were placed within a hydration chamber during this time. Following overnight incubation, the slides were then washed in 0.2% Triton-X 100 in PBS for 10 min twice and washed in PBS for 10 min twice. Secondary antibody solution was prepared similar to the primary antibody solution with secondary antibody diluted following manufacturer recommendations in PBS with a final concentration of 0.5% BSA. Slides were incubated with secondary antibody solution away from light for 45 min at room temperature and then washed in 0.2% Triton-X 100 in metal-free PBS for 10 min, twice, and then in metal-free PBS for 10 min, twice. Slides were then treated with 3-uM DAPI solution for 2 min away from light and subsequently washed in metal-free PBS for 10 min. Tissues were then mounted with coverslips using Vectashield Plus Antifade Mounting Medium (Vector Laboratories) and sealed with clear nail polish (Revlon 771). Tissues were imaged on Zeiss LSM 880 with oil immersion.

Antibodies with successful positive staining were subsequently conjugated to lanthanide metals (Additional file 1: Table S3) following the protocol associated with the Maxpar X8 Antibody Labeling Kit (Fluidigm).

### Imaging mass cytometry

Dura and tumor samples frozen in O.C.T. at −80°C were thawed, removed from O.C.T., and immediately fixed in 10% neutral buffered formalin for 22 h. Samples were then transferred to 70% metal-free ethanol, embedded in paraffin wax, and sectioned at a thickness of 5 um onto standard slides. Slides were baked in an oven at 56°C overnight to melt the paraffin wax and then placed in xylene for 20 min and then rehydrated in the following metal-free solutions of ethanol for 5 min each: 100%, 100%, 95%, 95%, 80%, 80%, 70%, and 70%. After rehydration, slides were placed in metal-free water for 5 min on an orbital shaker and then incubated in pH 9 IHC Antigen Retrieval Solution (Invitrogen) at 96°C for 30 min. Slides were cooled in the antigen retrieval solution for 10 min at room temperature and washed in metal-free water for 10 min and metal-free PBS for 10 min. The tissues on the slides were outlined with a hydrophobic barrier pen (Liquid Blocker), and a solution of 3% bovine serum albumin (BSA) in PBS was placed on the tissues within the hydrophobic barriers for 45 min at room temperature. The lanthanide-conjugated antibody solution, with respective dilutions as outlined in Additional file 1: Table S3, was prepared in PBS with a final concentration of 0.5% BSA. This antibody solution was placed on the tissues within the hydrophobic barrier following removal of the 3% BSA solution. Slides were incubated with this antibody solution overnight at 4°C in a hydration chamber. Following overnight incubation, the slides were then washed in 0.2% Triton-X 100 in PBS for 8 min, twice. They were then washed in PBS for 8 min, twice. DNA-Intercalator (Fluidigm) solution was prepared in PBS at a dilution of 1:400 and incubated on the tissues within the hydrophobic barrier for 30 min at room temperature. Slides were washed in metal-free water for 5 min and then air-dried for 20 min. The prepared slides were imaged on the Hyperion System (Fluidigm). Imaging results were visualized through MCD Viewer (Fluidigm) and saved as 16-bit TIFF images. Individual channel intensities were manually selected and standardized throughout all images.

### Expression heatmaps and gene functional enrichment analysis

Expression heatmaps were generated by selecting the *n* most highly weighted genes in each of the top *m* PCs (*n* and *m* are indicated in the text corresponding to each heatmap). The expression of each gene was averaged within each cluster and scaled and the results were hierarchically clustered using heatmap2. Gene functional enrichment analysis was performed using ToppGene (https://toppgene.cchmc.org/enrichment.jsp) [24]. Hierarchically clustered gene groups were selected and the top one or two gene ontology biological pathways were displayed. All gene groups are listed in Additional file 2.

### Macrophage polarization, meningeal macrophage, and microglial scores

Macrophage polarization, meningeal macrophage, and microglial scores were generated using *AddModuleScore* (Seurat implementation) and previously published gene lists [10, 25, 26].

### Immunohistochemical staining of somatostatin receptor 2 and macrophage markers

Formalin-fixed, paraffin-embedded (FFPE) tissues were sectioned into 5-μm sections using a microtome and baked at 55-60°C for 2 h. FFPE sections were stained with hematoxylin and eosin (Thermo Fisher). Automated immunohistochemical staining was performed on the BOND Rxm (Leica Biosystems) on FFPE sections, using the Bond Polymer Refine Detection kit (DAB-based) for both mouse and rabbit primary antibodies (Leica Biosystems). Following baking and dewaxing, appropriate antigen retrieval was performed with citrate-based (ER1) or high-PH (ER2) buffers for 20 min. After endogenous peroxidase block and non-specific protein blocking (2.5% BSA with 5% goat serum in PBS), tissues were incubated in primary antibody for 60 min. Primary antibodies (diluted in blocking buffer) and dilutions used were as follows: rabbit Anti-Iba1 antibody [clone EPR16588] 1:200 (ab178846; Abcam), rabbit Anti-Mannose Receptor antibody 1:2000 (ab64693; Abcam), rabbit Anti-TMEM119 antibody-C-terminal 1:250 (ab185333; Abcam), rabbit Anti-Somatostatin Receptor 2 antibody [UMB1]-C-terminal 1:1000 (ab134152; Abcam), and mouse Anti-CD163 1:200 (NCL-L-CD163; Leica) (Additional file 1: Table S2). After polymer-based anti-rabbit or anti-mouse labeling with HRP, tissues were chromogenically developed with DAB for 10 min and counterstained with hematoxylin. Slides were dehydrated and mounted using xylene-based Cytoseal (Thermo Fisher).

### Image analysis of IHC of somatostatin receptor 2 and macrophage markers

Whole-slide scans were obtained on a Zeiss Axioscan Z1 brightfield slide scanner using a 20× objective lens. Whole-slide scans were analyzed using HALO software (Indica Labs) using the algorithm Area Quantification v2.1.11, quantifying the percent area positive for the indicated marker in the total analyzed area. Representative areas were also captured and assembled into panels using HALO software (Indica labs).

### TCR analysis

Raw TCR sequencing data was processed with the Cellranger V(D) J pipeline (10x genomics, default settings, version 2.0.0) mapped onto a human VDJ reference GRCh38-2.0.0. Clonotype analysis was performed using the scRepertoire R package [27]. All data processing was performed as outlined at https://github.com/ncborcherding/scRepertoire. The following clonotype states were as defined: hyperexpanded (50 < *X* ≤ 150), large (20 < *X* ≤ 50), medium (5 < *X* ≤ 20), small (1 < *X* ≤ 5), and single (0 < *X* ≤1), where *X* is the number of cells in which the clonotype appears.

### Copy number analysis

Copy number variants (CNVs) were assessed using the CONICSmat package for R [28]. Gene expression values were filtered and normalized as discussed at https://github.com/diazlab/CONICS. The *z*-score posterior probabilities were clustered, with a cut-off score of *z*=2, and cell barcodes from the ten clusters (MEN104), seven clusters (MEN108), and eight clusters (MEN09) were gathered and visualized on UMAP.

### Statistical analysis

Differential gene expression was calculated using the Wilcoxon rank-sum test (two-sided) as implemented in the Seurat R package. Bonferroni correction was used to adjust *p*-values based upon total number of features in the dataset. Enriched gene ontology biological pathways were assessed using ToppGene and the top one or two biological processes significant after Bonferroni correction were selected.

## Results

### The dura consists of diverse immune and non-immune cell types

**Fig. 1:**
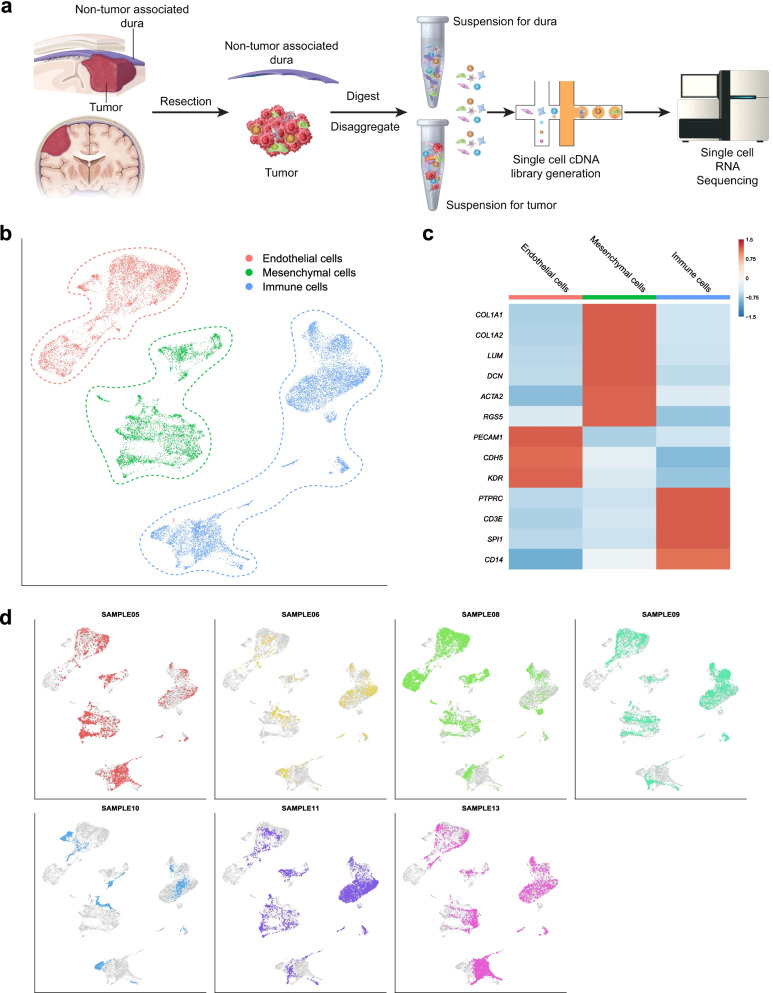
Single-cell preparation and sequencing shows diverse cell landscape of human dura. **A** Illustration of both non-tumor-associated dura and tumor resection and single-cell library preparation. **B** UMAP visualization of single-cell RNA-seq data identified by cell type. **C** Representative gene expression of select cell type gene markers. **D** UMAP visualization of single-cell RNA-seq data highlighting cells originating from each individual patient sample

To better understand the cellular composition of human dura, we performed scRNA-seq on samples of human dura and a subset of matched and non-matched primary meningioma samples derived from patients undergoing craniotomy for resection of intracranial meningiomas, which arise from the dura and thus are anatomically attached to this meningeal layer (Additional file 1: Table S1). In surgical resection of meningiomas, if possible, an adjacent region of dura grossly uninvolved with the tumor, as defined by the surgeon, is normally resected to ensure maximal tumor resection and reduce the risk of recurrence [19, 20]. This grossly uninvolved dura, which we define as “non-tumor-associated” dura, was subsequently harvested and used in our analyses. In total, seven dura samples and six primary meningioma samples (four matched and two non-matched) were dissociated and analyzed using scRNA-seq (Fig. 1A, Methods, Additional file 3). We first characterized the non-tumor-associated dura samples, performing unsupervised clustering and uniform manifold approximation and projection (UMAP) [22] analysis on 22,460 cells (Fig. 1B). Cells were initially classified into three cell populations using common markers for endothelial cells (*PECAM1*, *CDH5*, *KDR*), mesenchymal cells (*COL1A1*, *COL1A2*, *LUM*, *DCN*, *ACTA2*, *RGS5*), and immune cells (*PTPRC*, *CD3E*, *SPI1*, *CD14*) (Fig. 1C and Additional file 1: Table S2). The majority of cells were immune cells (10,423 cells), followed by endothelial cells (6283 cells) and mesenchymal cells (5754 cells). Each patient sample was represented in each of the three major cell types (Fig. 1D). These data demonstrate that the dura harbors a diverse cell population of both immune- and non-immune-derived cell types.

### Immune cell composition of non-tumor-associated dura

**Fig. 2:**
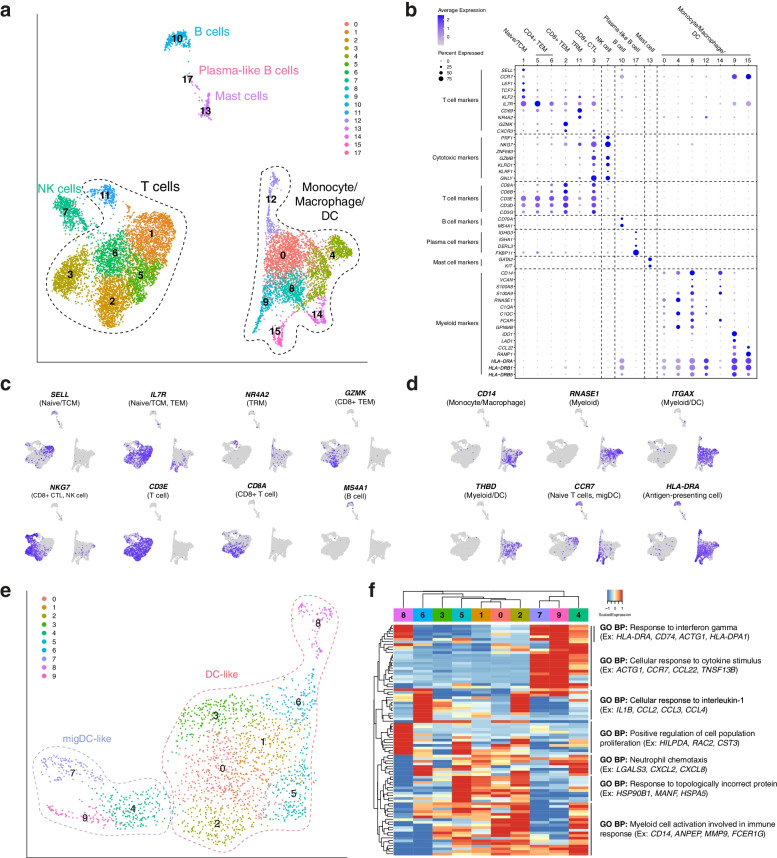
Immune cell composition of human dura consists of functionally diverse lymphoid and myeloid cell types. **A** UMAP visualization of immune cells identified by cell type (C1: naive/central memory T (TCM) cells; C5, C6: CD4+ effector memory T (TEM) cells; C2: CD8+ TEM cells; C11: resident memory T (TRM) cells; C3: CD8+ cytotoxic T cells (CTLs)). **B** Dot plot of gene expression of select cell type gene markers. **C**, **D** UMAP visualization of select lymphoid and myeloid marker gene expression. **E** UMAP visualization of dura DCs (C0, C1, C3, C4, C6, C7: DC-like; C2, C5, C8: migDC-like). **F** Expression heatmap of top 10 genes of top 10 principal components with hierarchical clustering and associated functional enrichment analysis of gene clusters

We next focused on resolving the immune cell (*PTPRC+*, which encodes CD45) landscape. To this end, we performed graph-based clustering and UMAP visualization on 10,423 immune cells (Fig. 2A). Clusters were characterized using a combination of previously reported markers and differentially expressed genes (Fig. 2B, Additional file 1: Table S2, Additional file 4) [29–38]. This revealed an appreciable population of lymphoid cells, including T cells, NK cells, B cells, and plasma-like B cells, as well as myeloid cells, including monocytes, macrophages, DCs, and mast cells. Expression of important marker genes for lymphoid and myeloid cells were visualized in the UMAP layouts for the indicated cell populations (Fig. 2C, D, respectively). T cells represented the majority of lymphoid cells observed (5458 cells) and included naive/central memory T (TCM) cells (1415 cells; which express *SELL*, *CCR7*, *LEF1*, *TCF7*, *KLF2*); CD4+ effector memory T (TEM) cells (1575 cells; *SELL*-, *CCR7*-, *IL7R*); CD8+ TEMs (1281 cells; *SELL-*, *CCR7-*, *IL7R*, *CD8A*, *CD8B*, *GZMK*^*hi*^, *CXCR3*^*hi*^); resident memory T (TRM) cells (251 cells; *CD69*, *NR4A2*, *IL7R*); and CD8+ cytotoxic T cells (CTLs) (936 cells; *PRF1*, *NKG7*, *ZNF683*, *GZMB*, *CD8A*, *CD8B*). Other lymphoid cell types identified included natural killer (NK) cells (571 cells; *PRF1*, *NKG7*, *GZMB*, *KLRD1*, *KLRF1*, *CD3-*), B cells (309 cells; *CD79A*, *MS4A1*, MHC class II+); and plasma-like B cells (35 cells; *IGHG3*, *IGHA1*, *DERL1*, *FKBP11*). Meanwhile, the monocyte/macrophage/DC population (3889 cells) was identified by monocyte-related (*CD14*, *VCAN*, *S100A8*, *S100A9*, MHC class II+), macrophage-related (*CD14*, *RNASE1*, *C1QA*, *C1QC*, *FCAR*, *GPNMB*), and DC-related (*CD14-*, *ITGAX*, *THBD*, and *IL3RA*) markers. As the cell identities of these clusters were not clearly identifiable by gene marker sets at this resolution, we initially defined this population as a general myeloid compartment composed of monocytes, macrophages, and DCs. Additionally, we identified mast cells (161 cells; *GATA2*, *KIT*, *HPGDS*) in the myeloid compartment. Finally, we sought to compare our data set to a previously published data set [10] that focused on immune cells in murine dura. We selected the top 20 differentially expressed genes (DEGs) for each general cell type in the mouse data set, identified their respective human homologues, and examined their expression in our human data set (Additional file 1: Fig. S1A, Additional file 5). Though not all genes had human homologs nor were expressed in the human data, we found specific expression of murine T/NKT, NK, migDC, and D-BAM markers in the corresponding human immune cell types. These data demonstrate that human dura is made up of a diverse population of immune cells, similar to murine dura, and cell-type-specific gene expression signatures are similar across species.

To better resolve the monocytes, macrophages, and DCs, we isolated all myeloid cells (excluding mast cells) for further analysis, including dimensionality reduction, clustering, and cell type annotation. We performed graph-based clustering and UMAP visualization on 3889 cells (Additional file 1: Fig. S1B), which revealed one monocyte/macrophage and two DC populations, DC-like and migratory DC-like (migDC-like), identified using marker gene sets (Additional file 1: Table S2, Additional file 1: Fig. S1C). Monocytes and macrophages were grouped together as these cells expressed varying levels of both marker gene sets. The low expression levels of markers previously associated with human microglia, such as *AIF1*, *C1QA*, and *GPR34* [26], and the expression of markers commonly associated with monocyte-derived macrophages suggested these cells were blood-derived rather than tissue resident. Interestingly, one cluster of cells (C6) was positive for monocyte and macrophage markers but lacked expression of MHC class II genes (*HLA-DRA*, *HLA-DRB1*), which suggested that they were myeloid-derived suppressor cells (MDSC-like) [38]. We similarly analyzed the expression of the top 30 DEGs expressed by matched myeloid cell types reported by Van Hove et al. [10] (Additional file 1: Fig. S1D, Additional file 5). Overall, we found that the monocyte/macrophage cluster expressed DEGs from each of the murine myeloid cell types, rather than specific cell types such as classical monocytes or D-BAMs. Notably, the MDSC-like cells under expressed most of these genes. Similarly, the DC-like cluster highly expressed DEGs from each of the murine myeloid cell types though C8 particularly expressed genes differentiating the murine cDC2 cell type. Finally, we found the migDC-like cluster specifically expressed genes identifying murine migDCs. Collectively, these data show the diverse repertoire of myeloid cells within the dura and some conservation across species.

We further characterized the DC population (DC-like: *CD14-*, *ITGAX+ CD1c-*, *THBD*^int^; migDC-like: *CD14- ITGAX- IL3RA+ CCR7+*) using unsupervised clustering and UMAP visualization of 2031 cells (Fig. 2E). To investigate variation within these cell populations, we hierarchically clustered the top 10 genes from each of the top 10 principal components (PCs) and used ToppGene [24] to characterize the functional enrichment of co-expressed genes (Fig. 2F, Additional file 2). This highlighted both the cell types present within the tissue, as well as the biological pathways associated with each cell type. DC-like clusters were characterized by the following pathways: “cellular response to interleukin-1,” “positive regulation of cell population proliferation,” and “response to topologically incorrect protein.” Meanwhile, migDC-like clusters were characterized by the following pathway: “cellular response to cytokine stimulus.” Shared pathways included “response to interferon gamma,” “neutrophil chemotaxis,” and “myeloid cell activation involved in immune response.” Interestingly, Chen et al. [33] and Pombo Antunes et al. [36] observed DC clusters similar to migDC-like cells that likewise differentially expressed genes such as *CCR7* and *LAMP3* and suggested that they are migratory DCs (migDCs) involved in immune cell recruitment. These data suggested that DCs harbored by the dura may be playing a role in establishing the dura immune microenvironment.

Collectively, our analysis demonstrated the presence of both lymphoid and myeloid cell subsets within the dura and underscored the dynamic nature of the dura immune microenvironment.

### Endothelial and mesenchymal cells comprise a significant proportion of cells in non-tumor-associated human dura

**Fig. 3:**
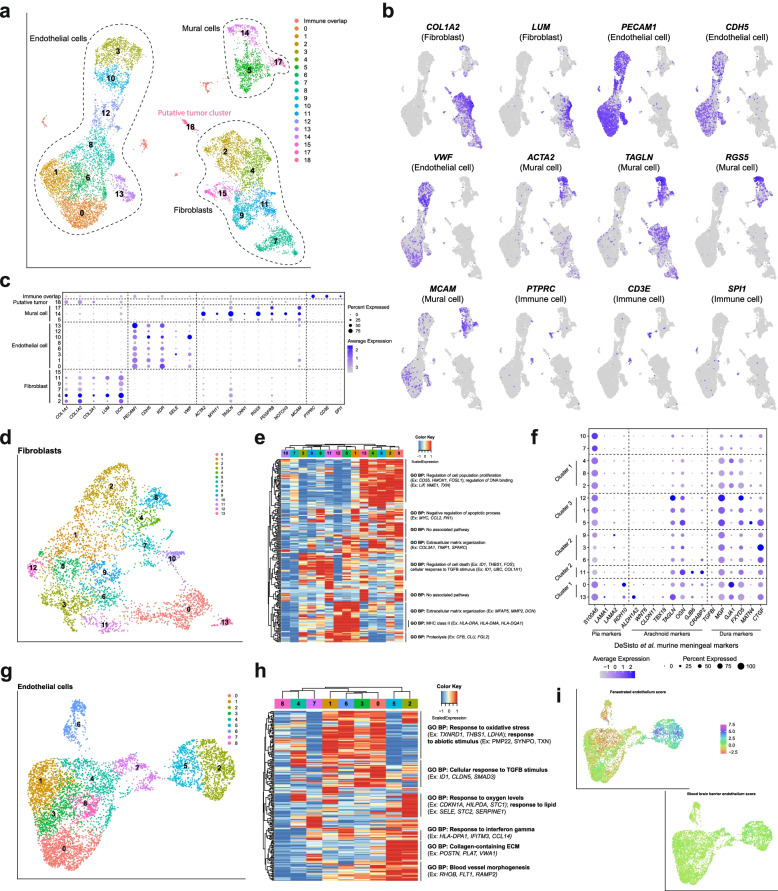
Functionally diverse non-immune cells comprise a significant proportion of human dura. **A** UMAP visualization of non-immune cells identified by cell type. **B** UMAP visualization of select endothelial, mural, and fibroblast markers. **C** Dot plot of representative gene expression of select cell type gene markers. **D** UMAP visualization of fibroblast endothelial cells. **E** Expression heatmap of top 15 genes of top 15 principal components of dura fibroblast cells with hierarchical clustering and associated functional enrichment analysis of gene clusters. **F** Dot plot of select meningeal fibroblast markers from Desisto et al. [39]. **G** UMAP visualization of dura endothelial cells. **H** Expression heatmap of top 15 genes of top 15 principal components of dura endothelial cells with hierarchical clustering and associated functional enrichment analysis of gene clusters. **I** UMAP visualization of fenestrated endothelium and blood-brain barrier scores

We next investigated non-immune (*PTPRC-*) cells by applying graph-based clustering and UMAP visualization followed by cell type annotation. This revealed three main cell types identified by previously reported marker gene sets and differentially expressed genes (Fig. 3A) [40–43]: endothelial cells (6157 cells; *PECAM1*, *CDH5*, *KDR*, *SELE*, *VWF*), fibroblasts (4132 cells; *LUM*, *DCN*, *COL1A1*, *COL1A2*, *COL3A1*), and mural cells (1368 cells; *ACTA2*, *MYH11*, *CNN1*, *RGS5*, *PDGFRB*, *NOTCH3*, *MCAM*, *CSPG4*) (Fig. 3B, C, Additional file 4). Though C18 (132 cells) was initially a small and indeterminate cluster, as we compared the matched dura and tumor datasets, further analysis revealed that C18 may represent a potential cluster of tumor cells originating from MEN08. Specifically, we observed the presence of copy number variants (CNVs) in 5q and 20q in addition to DEGs, such as *COL9A3* and *CRABP1*, shared with other putative tumor clusters described further below (Additional file 4, Fig. 7). Notably, no other samples harbored detectable populations that could include neoplastic cells. As non-tumor-associated dura is grossly observed to be separate from tumor, the presence of this population could represent an adjacent microscopic cluster of tumor cells. However, due to the limited number of samples and comparatively small number of observed tumor cells in general, we were unable to draw any significant conclusions. We also observed doublets (248 cells), defined by the co-expression of discordant markers for a mixture of cell types, although an increase in the number of transcripts was not detected. The presence of these cell types is consistent with our understanding of the gross structure of dura: a moderately vascularized tissue which harbors a collagen matrix scaffold which underpins the structure.

To better characterize the fibroblast cell population, we selected and analyzed these cells using graph-based clustering and UMAP visualization (Fig. 3D). We hierarchically clustered the top 15 genes of the top 15 PCs to identify biological programs (Fig. 3E, Additional file 2). We observed considerable heterogeneity in gene expression profiles and their associated biological pathways with three major hierarchical clusters arising. Cluster 1, which consisted of C0, C2, C4, C8, and C13, was enriched in genes related to “regulation of cell population proliferation,” “regulation of DNA binding,” and “negative regulation of apoptotic process.” These results suggested a distinct subpopulation of proliferating fibroblasts. Another cluster, characterized by C3, C6, C9, and C11, was enriched in genes related to “regulation of cell death” and “cellular response to TGFβ stimulus.” TGFβ stimulus has been shown to induce fibroblast activation and drive scarring in several organ systems [44, 45] and suggested perturbation of the fibroblasts. However, the cause of this activation could be attributed to many sources, such as biological phenomena occurring within the tissue or surgical excision. The third cluster, characterized by C1, C5, and C12, was enriched in genes related to “extracellular matrix organization,” “MHC class II,” and “proteolysis.” MHC class II upregulation has been shown to be induced by IFNγ in dermal fibroblasts [46], and signaling via MHC class II receptors has been shown to lead to cytokine secretion [47]. Based upon these results, these cells may have been actively responding to, and contributing to, immune regulation, in addition to ECM organization. These results highlighted the heterogeneity of fibroblasts in the dura and suggested functional specialization of specific subpopulations.

We next compared the fibroblast clusters to murine meningeal fibroblasts recently described by DeSisto et al. [39] (Fig. 3F). Specifically, we observed high expression of markers associated with murine dura fibroblasts, such as *MGP*, *GJA1*, and *FXYD5*, though not all markers, such as *TGFBI*, were highly expressed. We found that markers used to delineate different dura layers in mouse models, such as *MATN4*, *CTGF*, *NPPC*, and *CRABP2*, were not well represented in our data set [48]. Arachnoid markers *TAGLN* and *OGN*, also observed in murine dura fibroblasts, were highly expressed in our data set. Interestingly, although C0 and C13 co-clustered with C2, C4, and C8 based upon expression of PC genes (Fig. 3E), only C0 and C13 were enriched in both pia (*RDH10*) and arachnoid markers (*GJB6* and *CRABP2*). Similarly, C11 was more enriched in arachnoid markers (*GJB6* and *CRABP2*) than C3, C6, and C9 although they were hierarchically clustered together. These results suggested heterogeneity within hierarchical clusters and also that further study may be needed to understand the translation of some meningeal layer-specific murine markers to patient samples. This conclusion was supported by our investigation of the enrichment of murine leptomeningeal fibroblast-like cell (FLC) markers reported by DeSisto et al. [39] based upon data collected by Saunders et al. [49] (Additional file 1: Fig. S2).

We performed a similar analysis on the endothelial cell population by applying graph-based clustering and UMAP visualization (Fig. 3G) and hierarchical clustering of the top 15 genes of the top 15 PCs to identify enrichment of specific biological pathways (Fig. 3H, Additional file 2). Endothelial cells fell into three major hierarchical populations, with cluster 1 (consisting of C0, C1, C3, and C6) distinguished by its expression of genes related to “response to oxidative stress,” “response to abiotic stimulus,” and “cellular response to TGFβ stimulus.” As discussed previously, this may reflect endothelial cell biological phenomena or external stimulus, such as surgical excision or sample processing. Cluster 2, consisting of C2 and C5, were enriched in genes related to “response to oxygen levels,” “response to lipid levels,” “response to interferon gamma,” “collagen-containing ECM,” and “blood vessel morphogenesis.” These results suggested that cluster 2 is metabolically active and involved in vasculature development. Finally, cluster 3, which consists of C4, C7, and C8, exhibits low expression of these genes. As expected, we found that genes related to blood-brain barrier function were not enriched although fenestrated endothelium markers were enriched in hierarchical cluster two (Fig. 3I, Additional file 1: Table S2) [50, 51]. Dural fenestrated vessels have been reported and are important for molecule exchange with the blood [14, 52]. These data demonstrated evidence of a dynamic endothelial cell landscape in the dura layer composed of subpopulations with potentially different functions and the presence of fenestrated endothelium.

### Imaging mass cytometry of human dura

**Fig. 4:**
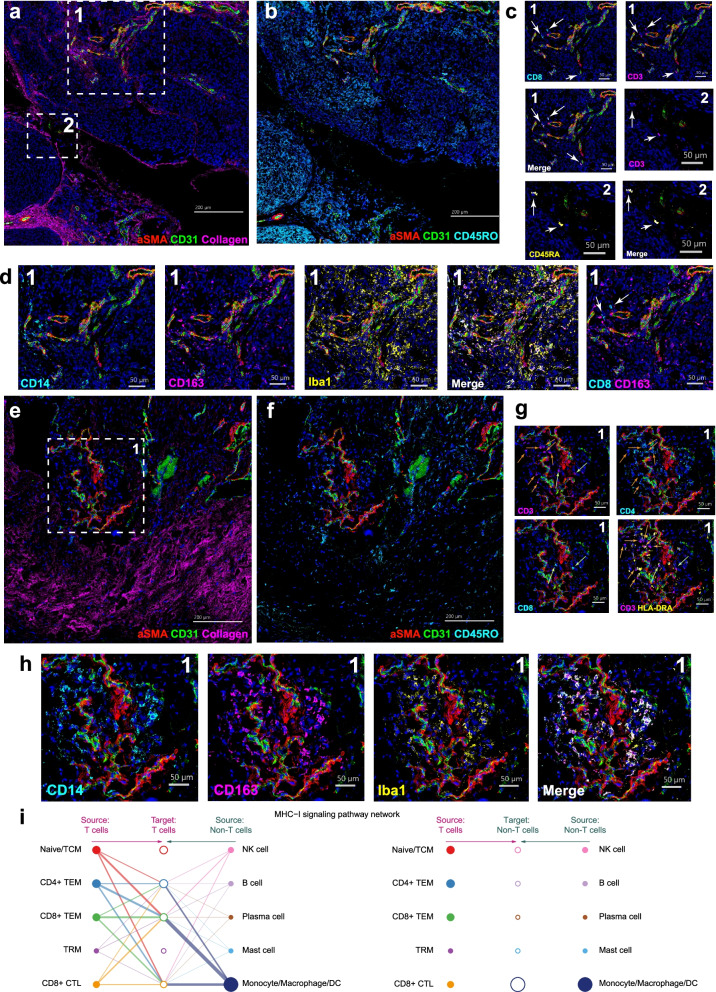
Imaging mass cytometry of a human dura sample reveals intricate spatial relationships among immune, endothelial, and mesenchymal cell types. **A**, **B** Imaging mass cytometry of human dura sample DURA02 labeled with markers specified and specific regions of interest (ROIs) highlighted by dashed white boxes. **C**, **D** Relative position of each image is denoted by marked number in the top left or right corner. Markers are specified and color coded. White arrows label cells of interest. **E**, **F** Imaging mass cytometry of human dura sample DURA05 with markers specified and the specific region of interest (ROI) highlighted by a dashed white box. **G**, **H** Relative position of each image is denoted by marked number at the top right corner. Markers are specified and color coded. Green arrows represent CD8+ T cells and orange arrows represent CD4+ T cells. **I** Hierarchical plot showing the inferred network for MHC-I signaling for immune cells from Fig. 2A. The left and right portions of the plot show autocrine and paracrine signaling to T cells and remaining immune cells, respectively. Solid circles represent the source of MHC class I ligands and open circles represent the target of said MHC class I ligands. Circle sizes are proportional to the number of cells and width of connecting lines indicate the communication probability of said interaction

Following characterization of human dura at single-cell resolution, we performed imaging mass cytometry on available dura samples, DURA02 and DURA05, to visualize the spatial relationship among these cell types. Similar to our approach with scRNA-seq, we sought to first identify the immune, endothelial, and mesenchymal cell types using specific cell markers (Additional file 1: Table S3). We identified vasculature by the presence of endothelial cells (CD31*+*, green), which was surrounded by vascular smooth muscle cells (a-SMA+, red) in sample DURA02 (Fig. 4A). Moreover, we observed diffuse presence of collagen (magenta) throughout the tissue as expected. Next, we focused on immune cells, observing concentrated regions of CD45RO expression (cyan) (Fig. 4B). Focusing on T cells, we observed in region 1 the presence of CD8 T cells based upon overlap of CD8 (cyan) and CD3 (magenta). Furthermore, we observed the presence of either naïve, or terminally differentiated, T cells with CD3 and CD45RA overlap. We also observed in region 1 the presence of cells with overlapping expression of CD14 and CD163, which may represent meningeal macrophages (Fig. 4D) [53–55]. Iba1, a common microglial marker that is lowly expressed in meningeal macrophages [53–55], showed some overlap with CD14 and CD163. However, Iba1+CD14-CD163- cells were likewise observed, suggesting a separate Iba1+ cell population harbored by the dura (Fig. 4D). CD8 T cells were observed to localize nearby CD163+ cells near the vasculature (Fig. 4D). Notably, the majority of these immune cells were observed close to, but outside of, defined CD31+ vasculature. Finally, we observed the presence of GZMB+ CD11b+ cells, often localized within CD31+ vasculature (Additional file 1: Fig. S3A), indicating circulation of cytotoxic immune cells within non-tumor-associated dura. Imaging of DURA05 demonstrated similar results (Fig. 4E–G) with clear CD31+ vasculature surrounded by vascular smooth muscle cells (Fig. 4E) though immune cells, labeled by CD45RO, were sparser and localized around the vasculature (Fig. 4F). Both CD8+ T cells (green arrows) and CD4+ T cells (white arrows) were observed in this sample in region 1 (Fig. 4G). Furthermore, we observed several CD4+ T cells to be adjacent to HLA-DRA+ cells. In contrast, we observed more overlap among CD14, CD163, and Iba1 (Fig. 4H) with fewer Iba1+CD14-CD163- cells. Finally, we observed GZMB+/CD11b+ cells mostly within CD31+ vasculature though infiltration beyond CD31+ vasculature was also noted (Additional file 1: Fig. S3B).

As cross-presentation of resident macrophages has been suggested [56], and we observed adjacent CD8+ T cells and CD163+CD14+ macrophages, we investigated whether genes associated with such pathways may be overrepresented in the single-cell data. Specifically, applying CellChat [57], which infers and analyzes intercellular signaling pathways, to the immune cell population from Fig. 2A, we identified several signaling pathways that were significantly represented by the single-cell data (Additional file 6). In particular, the monocyte/macrophage/DC population was the most prominent and significant source of MHC-I-related ligands targeting the various T cell populations, with the exception of resident memory T cells (left side of Fig. 4I). Some autocrine signaling was observed with CD4+ TEM cells, CD8+ TEM cells, and CD8+ CTLs. Furthermore, as expected, no significant relationships were observed with non-T cells as the target (right side of Fig. 4I). While these data raised the possibility that interaction between APCs and T cells may occur within the dura tissue itself, further investigation will be required to fully understand the functional roles of these immune cells within the dura as our current conclusions are limited due to the low number of samples and sites of imaging.

### Distinct gene expression profiles demarcate immune cells infiltrating meningiomas from those in non-tumor-associated dura

**Fig. 5:**
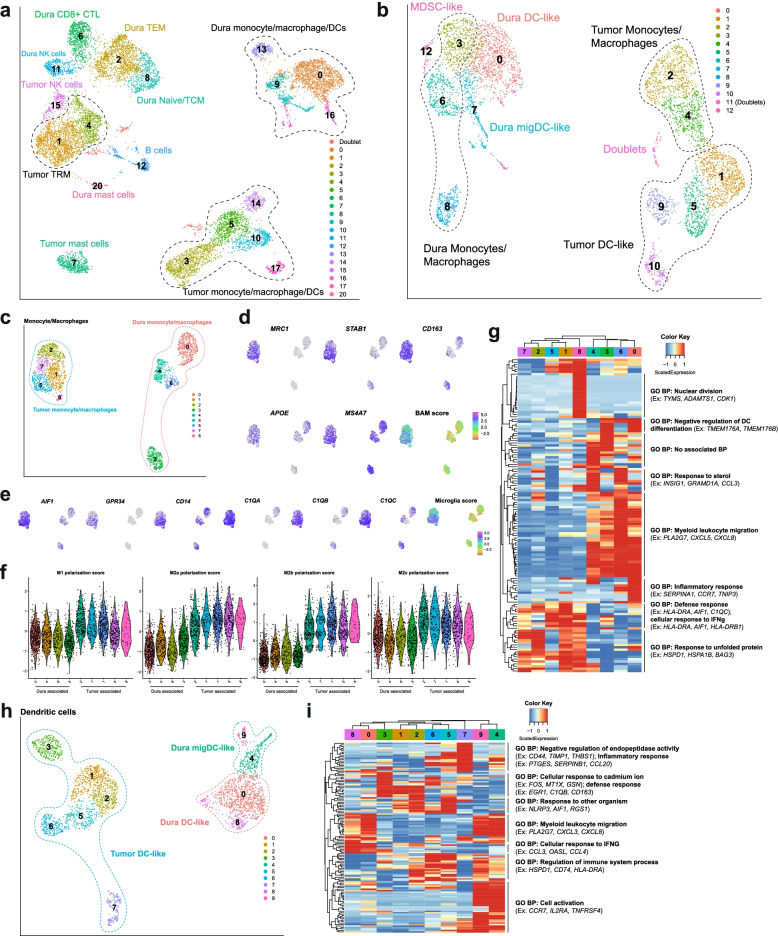
Non-tumor-associated human dura and meningioma tumor samples show distinctively different immune cell populations. **A** UMAP visualization of dura and tumor immune cells identified by cell type. **B** UMAP visualization of dura and tumor monocyte/macrophage/DCs identified by cell type. **C** UMAP visualization of dura and tumor monocyte/macrophages identified by cell type. **D** UMAP visualization of select border-associated macrophage (BAM) gene markers and aggregated score. **E** UMAP visualization of select microglial gene markers and aggregated score. **F** Violin plots of M1, M2a, M2b, and M2c macrophage polarization state scores of each cluster from Fig. 5C. **G** Expression heatmap of the top 15 genes of the top 10 principal components with hierarchical clustering and associated functional enrichment analysis of gene clusters. **H** UMAP visualization of dura and tumor DCs identified by cell type. **I** Expression heatmap of the top 15 genes of the top 10 principal components with hierarchical clustering and associated functional enrichment analysis of gene clusters

In a subset of patients in our cohort, we were able to collect matched meningioma samples together with non-tumor-associated dura (Additional file 1: Table S1). We analyzed the immune cells of four paired meningioma and non-tumor-associated dura samples composed of 12,581 cells and used the same markers described above to identify cell types. Within each cell type, we observed clear differences in cell state between cells isolated from each location (Fig. 5A, Additional file 1: Fig. S4A, Additional file 1: Table S2, Additional file 4). Notably, we observed that T cells, NK cells, monocytes/macrophages/DCs, and mast cells cluster separately based on tissue origin, whereas B cells from both dura and tumor clustered together. Though dura T cells consist of naïve/TCM cells, TEM cells, and CD8+ CTLs, only TRM cells were observed in the tumor samples.

Comparing top DEGs which differentiate dura-originating from tumor-originating T cells, dura T cells’ DEGs were related to T cell migration and function, such as *CXCR3* [58] and *ITGAL* [59], as well as cell motility genes *SUSD3* and *FGD3* [60, 61] (Additional file 7). In contrast, tumor T cells’ DEGs coded for heat shock proteins, such as *HSPA6*, *HSPA1A*, and *HSPA1B* in addition to genes related to T cell development and function, such as *NR4A1* [62, 63] and *NR4A2* [62]. Tumor NK cells expressed similar DEGs to tumor T cells and were enriched for genes associated with protein folding and cytokine expression, including genes coding for heat shock proteins *HSPA6*, *HSPA1B*, and *HSPA1A*, and *IFNG*, a common cytotoxic marker (Additional file 7). However, dura NK cells were enriched for genes that are associated with NK effector function, such as *SH2D1B* [64] and *KLRF1* [65]*.* Interestingly, *NLRC3*, a negative regulator of the innate immune response [66], was also overexpressed. Collectively, these data suggested that T cells and NK cells might have different functions in immune regulation depending on tissue of residence. However, as mentioned previously, although both types of tissues were processed similarly, the presence of heat shock proteins may indicate differing responses to the dissociation process rather than reflecting differing cell states within respective tissues. Further investigation will be required to elucidate the clinical implications of these differences.

To further explore the differences among dura and tumor monocytes, macrophages, and DCs, we isolated and reanalyzed both dura and tumor myeloid clusters (excluding mast cells) (Fig. 5B). Marker gene sets were used to differentiate monocyte/macrophages, DC-like, and migDC-like cell clusters (Additional file 1: Table S2, Additional file 1: Fig. S4B). We isolated first dura and tumor monocyte/macrophage clusters and reanalyzed them (Fig. 5C). We used markers associated with microglia and border-associated macrophages (BAMs) in murine models (Additional file 1: Table S2) to determine the potential origin(s) of these tumor monocyte/macrophages [10, 26] (Figs. 5D, E). Both microglial and BAM markers were enriched in tumor-only clusters, and not in dura-only clusters, which suggested that these macrophages were tissue-resident and originated from either the dura or brain parenchyma rather than blood. Similar results were observed via immunohistochemical staining of one matched pair of non-tumor-associated dura and meningioma. Non-tumor-associated dura showed no presence of somatostatin receptor 2 (SSR2), a sensitive marker for meningioma tumor cells [67, 68], low levels of Iba1 (0.228% positive area), CD206 (0.964%), and CD163 (0.368%), and moderate levels of TMEM119 (7.74%), a selective marker for microglia in the brain parenchyma [69] (Additional file 1: Fig. S5). Meanwhile, matched tumor sample showed high levels of SSR2, Iba1 (26.5%), and TMEM119 (39.5%), and moderate levels of CD206 (8.81%) and CD163 (11.9%). Although these results suggested tumor samples contain higher levels of markers associated with BAM and microglia, given the limited number of samples and scope of this study, additional studies will be needed to determine the origin of these macrophages. This limitation of our study was further highlighted by minor discrepancies based on scRNA-seq data, IHC staining, and IMC staining as within non-tumor-associated dura tissue, a considerable population of CD163+ cells and Iba1+ cells were observed via IMC (Figs. 4D, H). Meanwhile, in the IHC staining, we observed low levels of CD206, CD163, and Iba1 expression in non-tumor-associated dura (Additional file 1: Fig. S5). Finally, in the scRNA-seq data, we observed in dura monocyte/macrophages very low levels of *MRC1* (which encodes CD206) and heterogenous expression of *CD163* and *AIF1* (which encodes Iba1), with high expression of these genes in a small population of cells (Fig. 5D, E). Potential reasons for these discrepancies include heterogenous populations of immune cells in non-tumor-associated dura that we were unable to capture with the low number of samples in our data set. Furthermore, as mentioned previously, dura is currently categorized as non-tumor-associated from a gross perspective and the proximity of the non-tumor-associated dura edge to the dural-based tumor mass may vary from case to case. Finally, a difference in mRNA and protein levels, lack of sensitivity to detect rare transcripts, and alterations in cell state may be contributing factors. As we further discuss, additional studies will be required to rigorously define non-tumor-associated dura both from an anatomical and cellular assessment. We then characterized the potential functionality of these macrophages by first assessing the macrophage polarization states in both dura and tumor clusters. Previously reported markers were aggregated to generate scores for M1, M2a, M2b, and M2c polarization [25] (Fig. 5F, Additional file 1: Table S4). Overall, we observed similar M2c scores between dura and tumor clusters. Interestingly, tumor clusters demonstrated marked elevation of both M1 score, which is associated with a pro-inflammatory immune environment, and M2a and M2b scores, which are associated with an anti-inflammatory immune environment [25]. Finally, we hierarchically clustered the top 15 genes of the top 10 PCs to infer biological pathways and determine whether they were distinct based upon tissue source (Fig. 5I, Additional file 2). Overall, we observed a difference in gene expression between dura monocyte/macrophages and tumor monocyte/macrophages with respect to biological pathway enrichment. Most prominently, all dura clusters were enriched for genes associated with “response to sterol” and “myeloid leukocyte migration,” with some clusters enriched in genes associated with “inflammatory response” and “negative regulation of DC differentiation.” Meanwhile, all tumor clusters were enriched for genes associated with “defense response,” “cellular response to IFNγ,” and “response to unfolded protein.” In particular, MHC class II genes such as *HLA-DRA* and *HLA-DRB1* were also upregulated by tumor-specific clusters. Overall, these results indicated that monocyte/macrophages in the dura may have different origins and functional profiles compared to those found in the tumor site.

Both dura and tumor DCs were similarly separated and reanalyzed (Fig. 5H, Additional file 1: Table S2). We analyzed the top 15 genes of the top 10 PCs to infer biological pathways, which revealed considerable heterogeneity (Fig. 5H, Additional file 2). Notably, all dura DC clusters, both migDC-like and general DC-like, were enriched in genes related to “myeloid leukocyte migration.” General dura DC-like clusters (C0 and C8) were enriched in genes related to “cellular response to IFNγ” while migDC-like clusters (C4 and C9) were enriched in genes related to “cell activation,” such as *CCR7* and *IL2RA*, and “regulation of immune system process.” Tumor-specific DC-like cluster C7 was enriched in genes related to “negative regulation of endopeptidase activity” and “inflammatory response” while C3 was enriched in genes related to “cellular response to cadmium ion” and “defense response.” Clusters C1, C2, C5, and C6 were enriched in genes related to “response to other organism” and “regulation of immune system process.” Dura and tumor DCs also have distinct expression profiles, with dura DCs enriched for genes related to cell migration and cell activation and tumor DCs enriched for genes that help mount an immune response.

Given that several studies have demonstrated a functional role for the meninges in CNS immunosurveillance in murine models [14, 70, 71], these comparisons between tumor- and dura-derived immune cells may reflect a similar role for the dura in human disease. However, given that meningioma arises from the meninges itself, another explanation may be that the dura is simply the tissue site through which immune cells migrate to the tumor. Further studies, especially of dura collected from patients with intraparenchymal tumors, will be required to better understand the role of the meninges in response to disease in humans.

### TCR analysis of human dura and meningioma samples

**Fig. 6:**
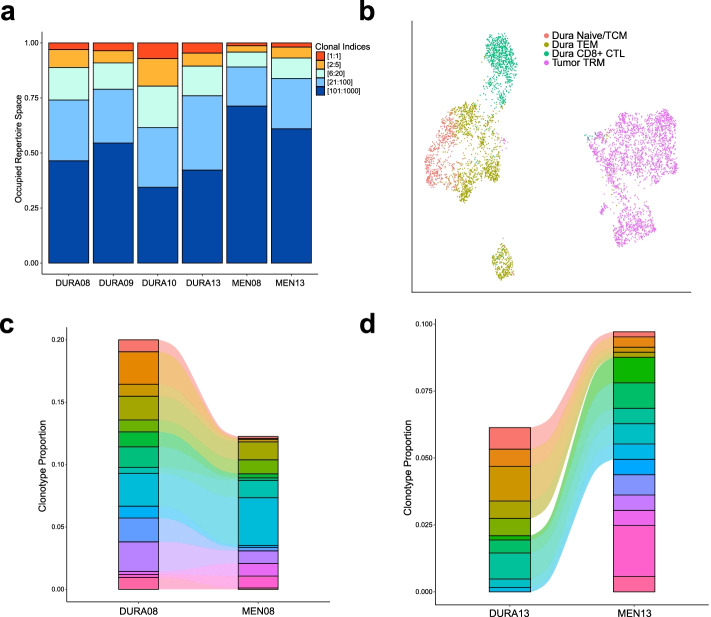
TCR frequency and expression overlap in paired non-tumor-associated dura and meningioma samples. **A** Clonal frequency of the dominant TCR, designated by absolute count, and groupings of TCRs ranked by absolute count. **B** UMAP visualization of dura and tumor T cells identified by cell type. **C**, **D** Alluvial plot demonstrating overlap of the top 15 and 16, respectively, TCRs in paired dura and meningioma samples ranked by relative frequency

To understand T cell clonotypic diversity within both matched dura and meningioma samples, we performed single-cell sequencing on V(D) J region enriched libraries from four dura samples and two matched meningioma samples (Additional file 1: Table S1). We first analyzed the relative frequency of T cell receptors (TCRs) by segregating the predominant clonotype (clone 1) from the rest, which were grouped based upon absolute count (i.e., clones 2–5, clones 6–20, clones 21–100, and clones 101–1000) (Fig. 6A). We observed a greater expansion of the top 20 clonotypes in the dura samples relative to those in the meningioma samples.

Following unsupervised clustering and UMAP analysis of SAMPLE08 and SAMPLE13 T cells alone from Fig. 5A, we observed a clear segregation of dura T cells from tumor T cells (Fig. 6B). Moreover, we generated alluvial plots of the top 15/16 TCRs, ranked by relative frequency with respect to each sample and represented by a distinct color, among the two paired dura and meningioma samples to determine the overlap of TCR presence (Fig. 6C, D, respectively). Strikingly, in the DURA08/MEN08 pair, all top 15 TCRs were identified at varying levels of expansion in both dura and tumor (Fig. 6C). Furthermore, DURA08 and MEN08 had a Morisita index, a measurement of the overlap between two data sets, of 0.484 when comparing the entire TCR repertoires of both samples, indicating a considerable amount of TCR overlap between the two samples (Additional file 1: Fig. S6). In the DURA13/MEN13 pair, 9 of the top 16 most frequently expressed TCRs were present in both DURA13 and MEN13 samples (Fig. 6D). DURA13 and MEN13 have a Morisita index of 0.13, indicating a smaller, but non-zero, overlap of all TCRs compared to the MEN08 and DURA08 pair (Additional file 1: Fig. S6). These data illustrated the T cell clonotypic diversity within the meninges and matched meningioma samples and reveal that TCR clonotypes can be present within both meningiomas and nearby, non-tumor-associated dura sites.

### Single-cell analysis demonstrates CNV heterogeneity in meningioma

**Fig. 7:**
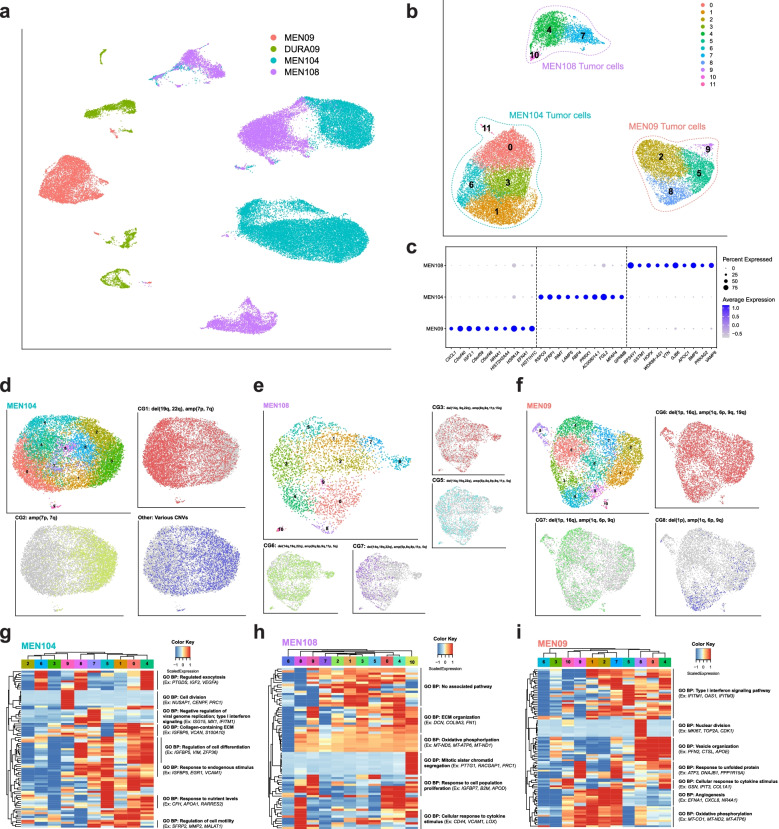
Analysis of meningioma cells reveals subclonal tumor populations with varying chromosomal abnormalities. **A** UMAP visualization of one paired dura and meningioma sample and two additional meningioma samples. **B** UMAP visualization of predicted tumor cells from MEN104, MEN105, and MEN09 samples. **C** Top 10 DEGs expressed in >50% cells of each sample-specific tumor cluster. **D-F** UMAP visualization of each individual sample-specific tumor cluster with unsupervised clustering and highlighted by respective CNV group (CG#) identities. **G–I** Expression heatmaps of the top 10 genes of the top 10 principal components of sample-specific tumor cells (**D-F**) with hierarchical clustering and associated functional enrichment analysis of gene clusters

In addition to analysis of the immune cells from paired dura and meningioma samples, we also performed copy number variant analysis on dura and meningioma pairs to identify putative tumor cells using the R package CONICSmat [28]. Initially, we identified tumor cells in only one paired sample (SAMPLE09), suggesting that tumor cells may have been selected against by the dissociation conditions necessary for dura processing. Therefore, we sequenced two additional meningioma tumor samples (MEN104 and MEN108) dissociated with the Miltenyi human tumor dissociation kit (Miltenyi Biotec), which involves a shorter disaggregation period (Additional file 1: Table S1, Methods). Together, these tumors represented the three WHO grades (MEN104: grade I, MEN108: grade II, MEN09: grade III). DURA09, MEN09, MEN104, consisting of both CD45+ and CD45− fractions, and MEN108, consisting of both CD45+ and CD45− fractions, samples were analyzed with graph-based clustering and UMAP visualization (Fig. 7A, Additional file 1: Figs. S7A and S7B). Using matched immune cells as a reference for CNV detection, we identified a population of cells in each patient sample harboring several chromosomal abnormalities, which we inferred to be tumor cells (Fig. 7B, Additional file 1: Figs. S7A, S7B, and S8). Specifically, the most prominent copy number variants observed for MEN104 were deletion of 19q and 22q (del(19q, 22q)) and amplification of 7p and 7q (amp(7p, 7q)); for MEN108, del(14q, 19q, 22q) and amp(5p, 8q, 9p, 9q, 11p, 15q); and for MEN09 del(1p, 16q) and amp(1q, 6p, 9q, 19q) (Additional file 1: Fig. S8). The majority of these chromosomal abnormalities are consistent with previous observations in WHO grades I and II meningiomas [72]. In WHO grade III meningiomas, amp(16q) and del(6p) are more frequently reported, although del(16q) and amp(6p) have also been observed. Isolation of the tumor cells reveals three distinct clusters with unique DEGs (Fig. 7B, C, Additional file 4).

Following CNV and DEG analysis, we isolated, reanalyzed, and investigated sample-specific meningioma cells to better characterize the CNV heterogeneity at the single-cell level. Unsupervised clustering and UMAP analysis were performed in addition to visualization of specific groups of cells based on their respective CNV profiles (Figs. 7D–F). For the tumor cluster derived from MEN104, three major subclonal populations were observed: CNV group 1 (CG1) which contained del(19q, 22q) and amp(7p, 7q), CG2 which contained amp(7p, 7q) and may represent the founding clone, and “Other” which contained the remaining minor CGs consisting of various combinations of the CNVs (Additional file 1: Fig. S8A). Differential expression analysis was used to characterize the expression signature of each CNV group (Additional file 4). To characterize gene expression heterogeneity in the clustered data (Fig. 7D), we analyzed the expression of the top 10 genes of each of the top 10 PCs (Fig. 7G, Additional file 2). Notably, we observed that clusters associated with CG1 (C0, C1, C4, C5, C7, and C8) were enriched in genes associated with several biological pathways, such as “regulation of cell differentiation,” “response to endogenous stimulus,” “response to nutrient levels,” and “regulation of cell motility,” while clusters associated with CG2 (C2, C3, and C6) were under-enriched. These results suggest CG1 cells may be more metabolically active and may be undergoing differentiation to develop a metastatic state. We also observed a cluster of cells (C9) enriched in genes related to “cell division,” indicating an actively dividing subpopulation. Similar analyses were performed for MEN108 (Figs. 7E, H) and MEN09 (Figs. 7F, I). Investigation of MEN108, unlike with MEN104, revealed several CGs with overlapping clustering patterns as observed with UMAP visualization (Fig. 7E). Analysis of the top PC genes revealed less heterogeneity as compared to MEN104 (Fig. 7H, Additional file 2). Most clusters expressed genes related to “oxidative phosphorylation” and “ECM organization” with cluster-specific expression of “response to cell population proliferation” and “cellular response to cytokine stimulus.” One cluster (C10) was enriched in genes related to “mitotic sister chromatid segregation,” suggesting an actively proliferating subpopulation of cells as observed in MEN104. Finally, analysis of MEN09 revealed one CG that characterized the majority of the cells and two smaller CGs (Fig. 7F). Overall, the majority of clusters showed high expression of genes related to signaling responses such as “type I interferon signaling pathway” and “cellular response to cytokine stimulus,” indicating a response of tumor cells to immune surveillance (Fig. 7I, Additional file 2). Several clusters were enriched for genes related to “angiogenesis,” an important process required for tumor development. Other pathways enriched included “oxidative phosphorylation” and “response to unfolded protein,” both of which suggested stress responses, and “vesicle organization.” As with the other tumor samples, an actively dividing cluster of cells was observed as C8 was enriched in genes related to “nuclear division.” Overall, analysis of these tumor cells, derived from tumors characterized by WHO grades I, II, and III, at single-cell resolution indicated the presence of CNV heterogeneity. These CGs were sometimes associated with particular gene expression patterns and respective functional profiles, as in MEN104. However, this was not ubiquitous as MEN108 and MEN09 did not exhibit CNV-associated gene expression profiles.

## Discussion

In this study, we presented the first comprehensive scRNA-seq analysis of human non-tumor-associated dura and primary meningioma tumor samples. The meninges have assumed growing importance in the study of CNS immunity and pathobiology as it has become clearer that they represent a dynamic microenvironment composed of unique cells with distinct immunologic, as well as non-immunologic, functions rather than a purely structural tissue barrier. To better understand this tissue site, we characterized both the immune and non-immune cell landscapes in non-tumor-associated dura in addition to analyzing both the gene and protein expression profiles of identified cell types using multiple platforms.

Building upon previous studies that characterized the immune microenvironment in murine dura [10, 14, 73], we observed a diverse collection of immune cells associated with both human non-tumor-associated dura and meningioma. The dura harbored several distinct T cell types ranging from T cells with a naive gene expression profile to those with cytotoxic profiles. The presence of these T cell subtypes may reflect ongoing immune surveillance by lymphocytes in non-tumor-associated dura, which has been demonstrated in murine models [14, 73]. Meanwhile, the majority of T cells in meningioma samples were TRM cells, which play a pivotal role in protective immunity and have been identified in many human solid cancers and brain infection [74]. Notably, the majority of TRM cells we observed were *CD8−* while current literature has focused on the role of *CD8*+ TRM cells in the immune response, especially against solid cancers. Furthermore, we observed an enrichment of BAM and microglial gene signatures, both of which are tissue-resident phenotypes, in tumor samples compared to non-tumor-associated dura samples and supported these findings with IHC staining. Although minor discrepancies in BAM signature expression were observed among the scRNA-seq, IHC, and IMC data, potential reasons include the heterogeneity of dura samples that was not captured by our limited number of samples, in addition to lack of correlation between mRNA and protein expression, transcripts that are not detected by scRNA-seq (“dropouts”), and potential cell state changes due to sample processing. BAMs have been described to play immune roles, such as support and maintenance of barrier function and surveillance of antigens, within the meninges [75] and microglia have been implicated in brain parenchyma homeostasis [76]. For these reasons, further investigation will be required to understand the function of these cells in non-tumor-associated dura compared to those present in the meningioma samples themselves. The significant difference in gene expression and associated functional profiles of immune cells based upon tissue origin warrants further investigation, as these differences may indicate targetable pathways to decrease tumor growth and the likelihood of recurrence.

Previous investigation in murine dura has established the role of the sinus vasculature in murine dura in allowing homeostatic T cell surveillance, in addition to demonstrating interaction between T cells and APCs adjacent to the sinus [70]. Our imaging suggested similar interactions may be occurring in human non-tumor-associated dura as we observed co-localization of CD4+ T cells and HLA-DRA+ cells in addition to CD8+ T cells and CD163+ macrophages near CD31+ stained vasculature. In addition, we found within the single-cell data that the monocyte/macrophage/DC population was a prominent source of MHC-I-related ligands while the T cells were prominent sources of respective targets. However, rigorous future studies will be required to determine whether such interactions occur within human dura.

By analyzing TCR clonotype diversity, we observed that the majority of T cells with highly expanded clonotypes expressed a cytotoxic phenotype in non-tumor-associated dura samples. Notably, tumor infiltrating T cells were less expanded and did not exhibit a robust cytotoxic phenotype, again illustrating a distinction between these environments though we were limited by the number of samples. In contrast, there were shared T cell clonotypes between paired non-tumor-associated dura and meningioma samples. Because meningiomas arise from the dura, we suggested that tumor-specific T cells could enter meningiomas through the same blood vessels supplying the surrounding non-tumor-associated dura tissue [52, 77] following a priming event either within the dura or elsewhere. As a result of the presence of immune cell infiltrate in non-tumor-associated dura tissue observed via IMC and shared clonotypes in paired dura and meningioma samples, we suggested that our non-tumor-associated dura may not be representative of normal dura in a healthy patient. In fact, our data indicated that an immune response may be occurring within the dura tissue itself. However, further investigation will be required to understand the mechanism behind T cell response to meningiomas, though the existence of shared T cell clones underscore the involvement of the dura in this process.

Meanwhile, investigating non-immune cells in human non-tumor-associated dura, we observed the presence of endothelial cells, fibroblasts, and mural cells, each of which plays an important role in the maintenance of the dura layer. The dura mater contains an abundant anastomotic arterial network [78], and in our samples, we observed an abundant presence of endothelial cells that were enriched in genes related to “blood vessel development” and “regulation of cell population proliferation.” Furthermore, as previously described, we observed the presence of fenestrated endothelium markers and a lack of blood-brain barrier related markers [14]. The presence of mural cells, which have been shown to be present and regulate vascular diameter and blood flow in the CNS [50], was also observed in our samples. However, while the distinction between pericyte and vascular smooth muscle cell can be made based upon immunostaining techniques [79], this distinction is difficult to make based upon specific gene markers. As a result, further studies will be required to determine specific vasculature location and correlated gene expression profiles. Finally, fibroblasts have been shown to play an important role in CNS development [1] and may even contribute to nociceptive signaling in murine models [80]. In our human dura samples, we observed an abundant presence of fibroblasts that contained a diverse gene expression profile enriched in biological pathways mainly concerning ECM development and response to various stimuli. These results indicated a dynamic cellular population in the human meninges, even beyond the development phase, that requires further investigation to better characterize and understand its role in maintaining dura mater homeostasis beyond a purely structural function. We also showed expression of dura-specific fibroblast markers generated from murine models [39], suggesting cross-species conservation.

Finally, from our analysis of patient-specific meningioma tumor cell populations, in which all three WHO grades were represented, we observed varying levels of CNV heterogeneity and heterogeneity of gene expression profiles across all samples. For one sample, we found CNV heterogeneity to be associated with specific gene expression signatures and associated biological pathways, while for the other two, such associations were not observed. Given our low number of samples taken at one time point, further studies are needed to understand the development of meningiomas and the relationship among these subclonal tumor populations. Specifically, with more samples, we can better determine the relationship among CNV heterogeneity, gene expression, and functional properties of meningioma cells. Interestingly, such transcriptomic intratumoral heterogeneity, in addition to epigenetic heterogeneity, has been associated with high-grade meningiomas at a bulk-tissue level with accompanying single-cell investigation of human cerebral organoids [81]. However, beyond this, investigation into primary meningioma tumor samples at a single-cell resolution has been relatively unexplored.

Our study provides the first scRNA-seq characterization of both immune and non-immune cell types in human non-tumor-associated dura and primary meningioma tumor samples. Similar to collaborative efforts such as the Human Cell Atlas [82], these data are a resource for furthering our understanding of the cellular composition of human dura. However, we recognize that there are several limitations of our study. From a sample collection standpoint, non-tumor-associated dura is determined based upon gross assessment of the relative location of tumor and dura by the surgeon. This may lead to heterogeneity in dura samples and potential presence of microscopic tumor clusters, as observed in one of our samples. As a result, we recognize that our conclusions are limited to describing the composition and cell states within non-tumor-associated dura collected from patients with meningioma, which may or may not be similar to normal, homeostatic dura tissue or dura tissue from patients with non-tumor conditions. However, this caveat does not limit the implications of our work as we make important observations regarding both the immune and non-immune cell compositions of human dura and the potential roles they have in response to disease, building upon the work performed in murine models [10, 14, 36, 70]. We acknowledge that there are additional limitations including the number of samples in our study, potential bias due to sample preparation, and limited validation of cell type presence via IHC and IMC. Further studies will be required to develop our understanding of the role of dura in the context of meningioma in addition to comparing how this role may change in other tumor settings depending on the type of tumor present, such as intraparenchymal brain tumors like glioblastoma multiforme or brain metastases. Additional studies will also be required to investigate whether our current standard for resecting meningioma tumors, and their respective dura borders, can be improved by systematically investigating the cellular composition, and the respective cell states, of the dura based upon distance from the primary tumor. We also aim to investigate additional opportunities to explore this tissue site across the full extent of its anatomic locations in normal and non-tumor settings. Given the growing evidence implicating the importance of the dura in biological pathways, such as immunosurveillance of the CNS, and in particular brain tumors [17, 83], and its current relevance in practical medical settings, such as embolization of the middle meningeal artery for new or recurrent chronic subdural hematoma [18], we envision there to be significant translational and clinical implications of an improved understanding of the biology of the human dura in the context of disease.

## Conclusions

Our characterization of human non-tumor-associated dura and primary meningioma tumor samples suggests new roles of the human dura in the context of CNS immune surveillance and reveals CNV heterogeneity in meningioma. The identification of a diverse repertoire of immune cells and associated phenotypes in dura, imaging studies that suggest co-localization of T cells and APCs within the dura tissue, and overlapping TCRs between dura and meningioma samples suggest the presence of immunosurveillance in the dura. Meanwhile, investigation of non-immune-related cells suggests that the dura is a dynamic, and constantly developing, layer of tissue and demonstrates subclonal heterogeneity in primary meningioma. The implications of this study are important as first, they contribute to the growing consensus that the dura layer plays a pivotal role in CNS immunosurveillance. Just as importantly, our study provides evidence of such biological phenomena in human samples, providing a foundation for both pre-clinical models and future translational studies.

## Supplementary Information

**Additional file 1: **Figs. S1-S8, Tables S1-S4. **Fig. S1.** Analysis of human dura immune cells and comparison to murine dura immune cells. **Fig. S2.** Leptomeningeal fibroblast markers. **Fig. S3.** Imaging mass cytometry of CD11b+ GZMB+ immune cells in DURA02 and DURA05. **Fig. S4.** Gene markers for *PTPRC+* cells in dura and tumor samples. **Fig. S5.** Immunohistochemistry of a matched pair of non-tumor-associated dura and tumor. **Fig. S6.** Quantification of TCR overlap. **Fig. S7.** Analysis of DURA09, MEN09, MEN104, and MEN108 samples differentiated by general cell types. **Fig. S8.** CONICSmat analysis of putative patient specific tumor cells. **Table S1.** Patient demographics and sample characteristics. **Table S2.** Gene markers for cell type identification. **Table S3.** Antibody Information. **Table S4.** Gene markers for macrophage polarization states.

**Additional file 2:** Analysis of principal component genes of different cell types in dura and tumor samples and associated gene ontology biological pathways.

**Additional file 3:** Cellranger output of single cell RNA-sequencing sample quality metrics.

**Additional file 4:** DEGs of different Seurat objects

**Additional file 5:** Van Hove Markers

**Additional file 6:** Target-ligand interactions output from CellChat.

**Additional file 7:** Dura and tumor immune cell DEGs.

## Data Availability

All fastq files are available in the NCBI Sequence Read Archive (SRA) with the exception of V(D) J fastq files for two samples, DURA13 and MEN13, which are in bam file format (https://www.ncbi.nlm.nih.gov/bioproject/PRJNA826269) [84]. As V(D) J bam files generated by the Cellranger V(D) J pipeline (10x Genomics) are not accepted by the SRA, data for these samples are available on the open-access data sharing platform Zenodo (10.5281/zenodo.4932158) [85]. Processed gene expression and V(D) J matrices and Seurat objects used for all analyses are also available on Zenodo.
