# Supplementary material for: Single-cell profiling of human dura and meningioma reveals cellular meningeal landscape and insights into meningioma immune response
Source: Genome Med. 2022 May 10;14:49. doi: 10.1186/s13073-022-01051-9 (PMC9088131; doi:10.1186/s13073-022-01051-9)
Supplement: Supplementary file 1 — Additional file 1: Figs. S1-S8, Tables S1-S4. Fig. S1. Analysis of human dura immune cells and comparison to murine dura immune cells. Fig. S2. Leptomeningeal fibroblast markers. Fig. S3. Imaging mass cytometry of CD11b+ GZMB+ immune cells in DURA02 and DURA05. Fig. S4. Gene markers for PTPRC+ cells in dura and tumor samples. Fig. S5. Immunohistochemistry of a matched pair of non-tumor-associated dura and tumor. Fig. S6. Quantification of TCR overlap. Fig. S7. Analysis of DURA09, MEN09, MEN104, and MEN108 samples differentiated by general cell types. Fig. S8. CONICSmat analysis of putative patient specific tumor cells. Table S1. Patient demographics and sample characteristics. Table S2. Gene markers for cell type identification. Table S3. Antibody Information. Table S4. Gene markers for macrophage polarization states. [file 13073_2022_1051_MOESM1_ESM.docx]

**Additional File 1**

**
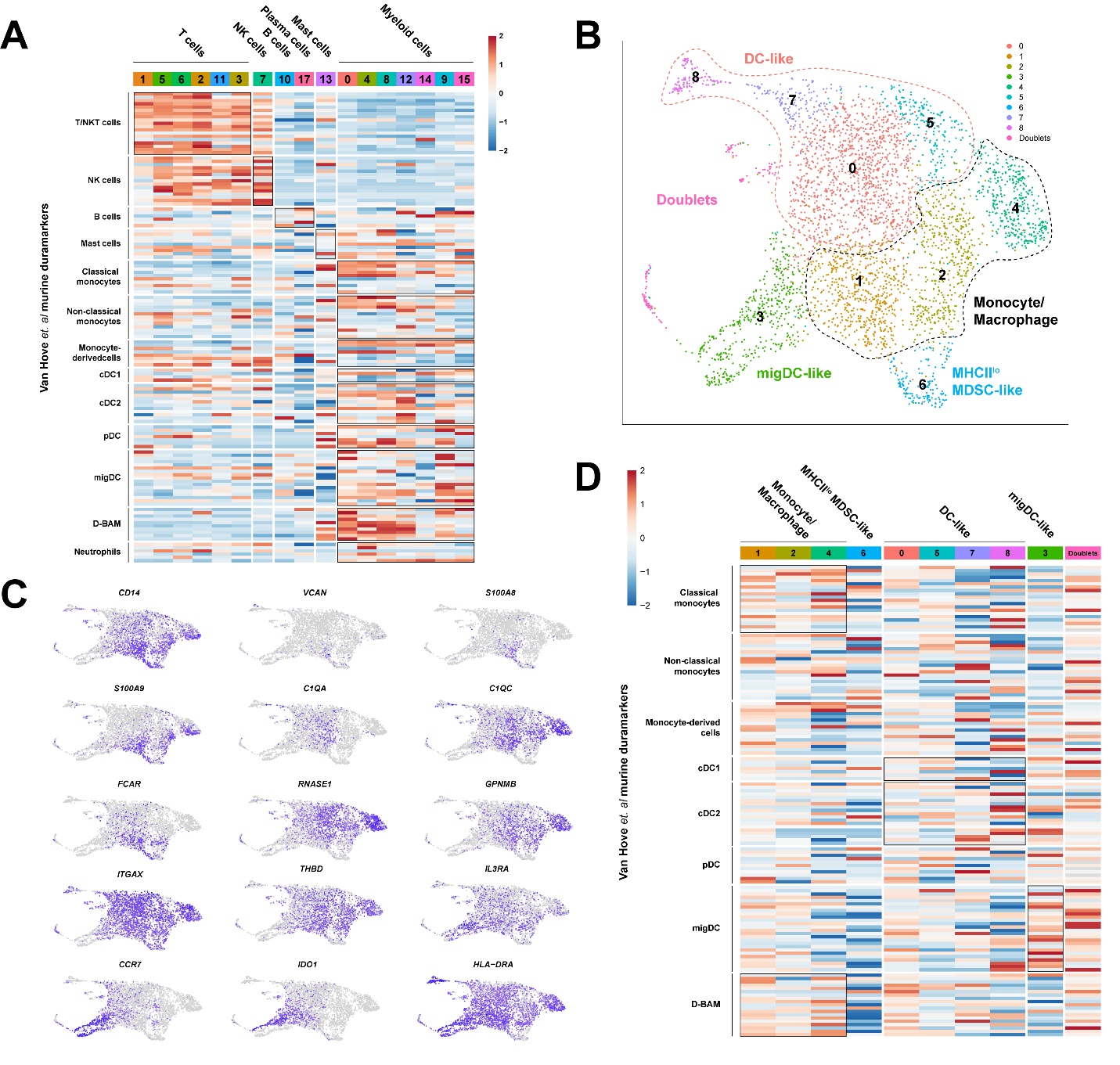
**

**Fig. S1.** **Analysis of human dura immune cells and comparison to murine dura immune cells.** **(A)** Heatmap visualization of expression of the top 20 DEGs of each cell type characterized by Van Hove *et al* (10)*.* **(B)** UMAP visualization of dura myeloid cells excluding mast cells. Monocyte/macrophages include C1, C2, C4. MHCII^lo^ MDSC-like cells include C6. DC-like cells include C0, C5, C7, and C8. migDC-like cells include C3. **(C)** UMAP visualization of dura myeloid cells excluding mast cells for select monocyte, macrophage, and DC marker genes. **(D)** Heatmap visualization of expression of the top 30 DEGs of each cell type characterized by Van Hove *et al* (10).


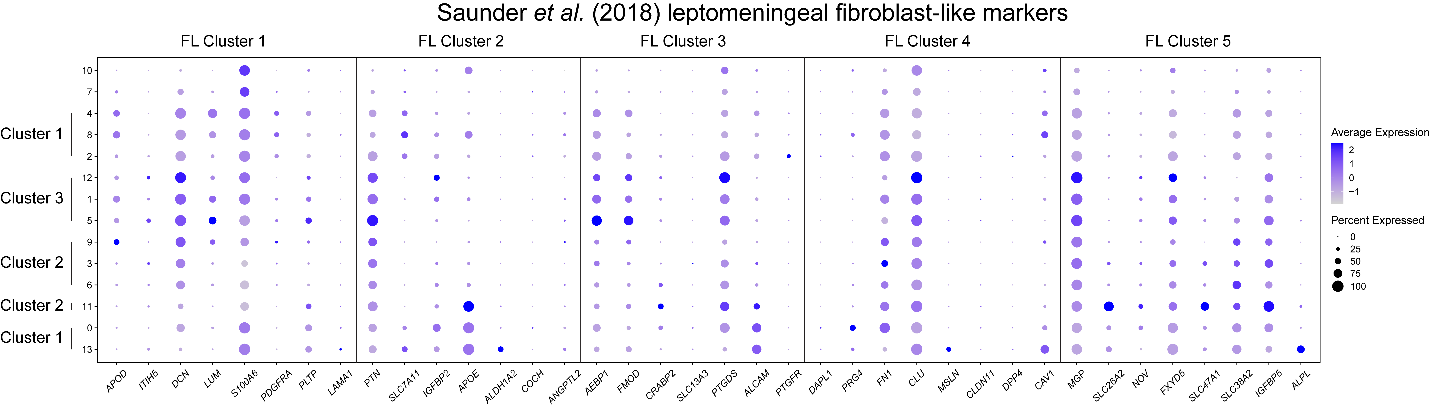


Fig. S2. Leptomeningeal fibroblast markers. Dot plot visualization of top DEGs of leptomeningeal fibroblast-like cell (FLC) clusters based upon the Saunders et al. (49) data set. Clustering of FLCs was performed and DEGs were reported by DeSisto *et al.* (39).

**
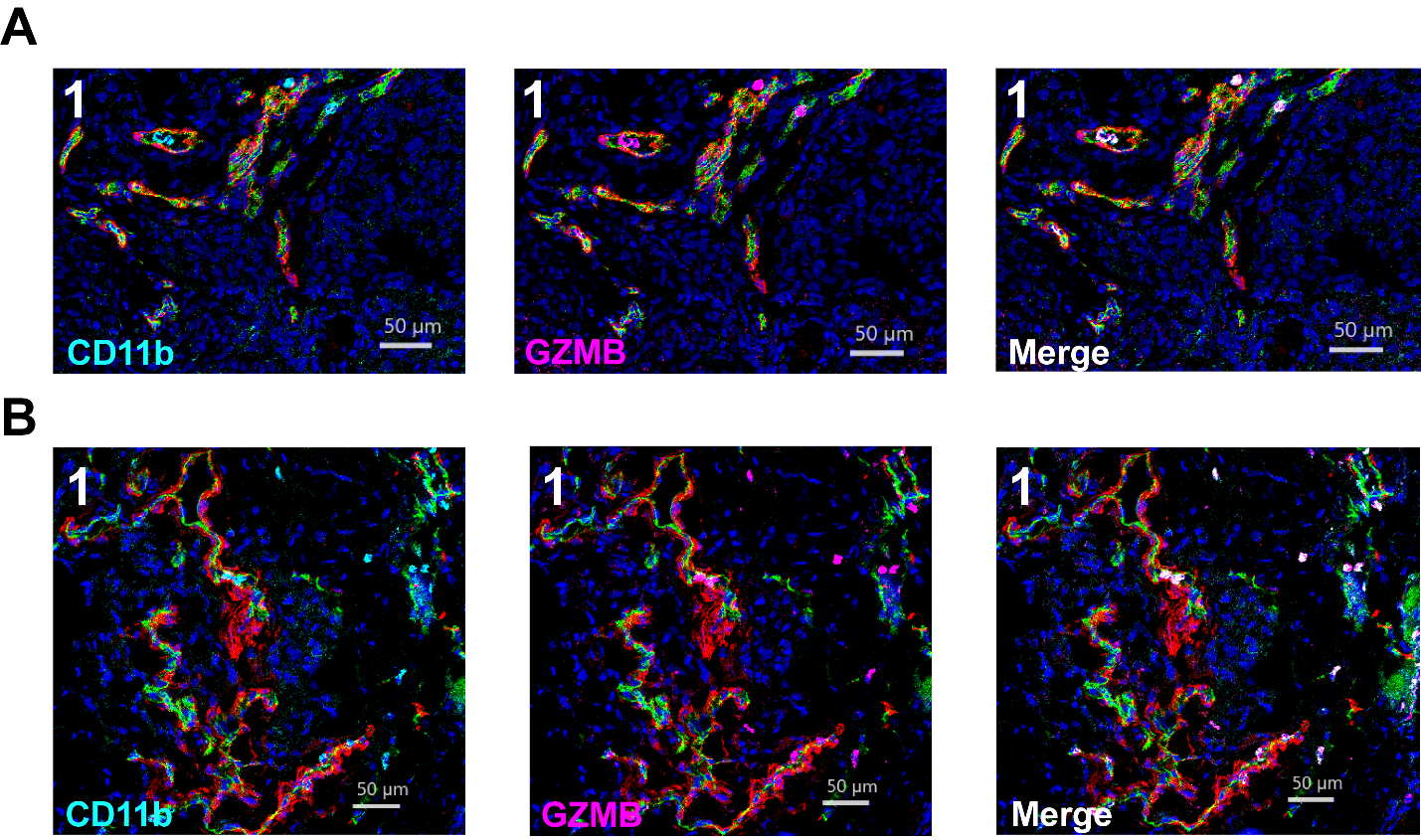
**

**Fig. S3. Imaging mass cytometry of CD11b+ GZMB+ immune cells in DURA02 and DURA05. (A, B)** Imaging mass cytometry of human dura sample DURA02 and DURA05, respectively, labeled with markers specified. Relative position of each image is denoted by bolded number in top left corner in reference to marked positions in Fig. 4A and Fig. 4E.


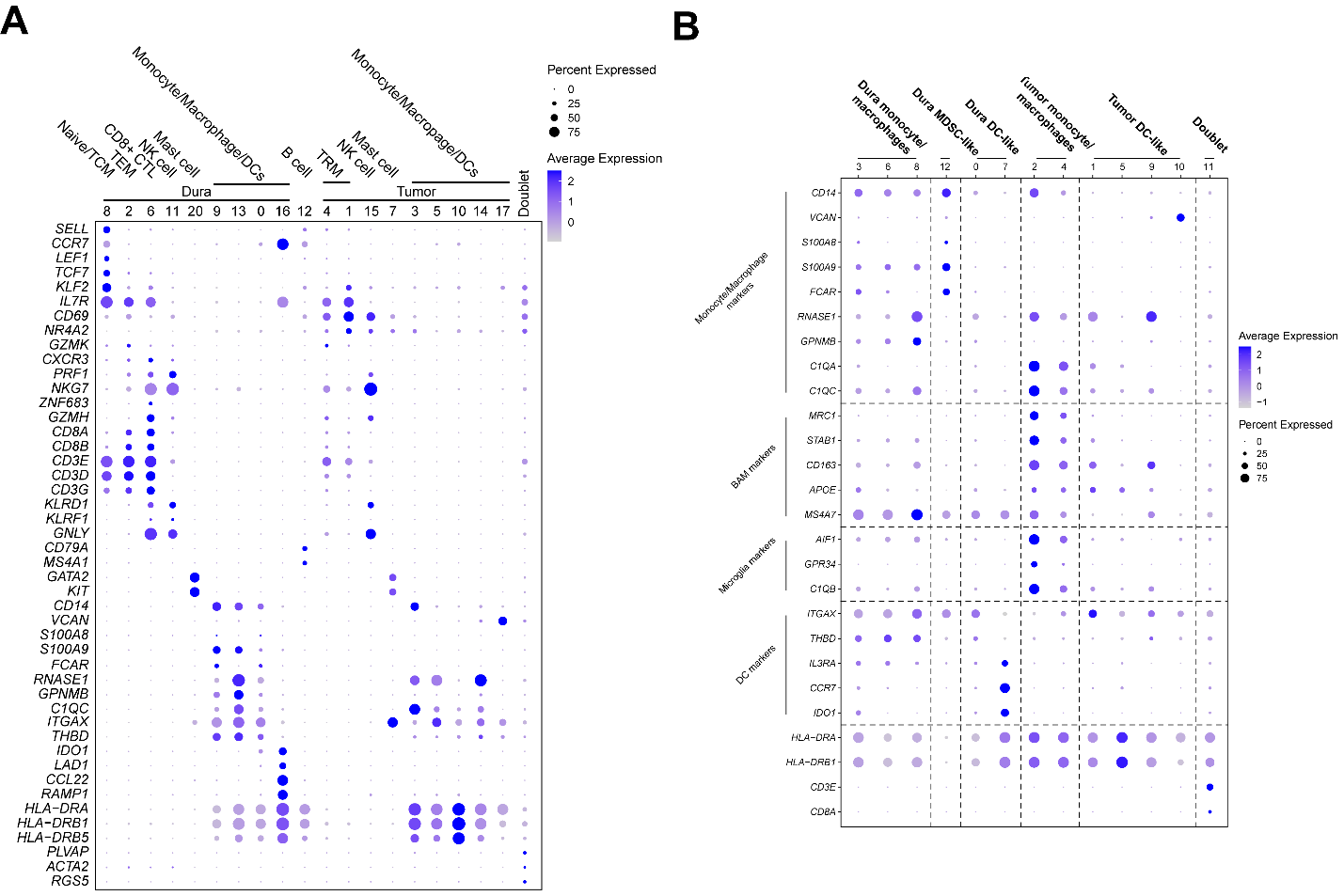


Fig. S4. Gene markers for *PTPRC+* cells in dura and tumor samples. (A) Dot plot visualization of gene markers used for cell identification of *CD45*+ cells in dura and tumor samples (TCM: central memory T cell; TEM: effector memory T cell; CTL: cytotoxic T cell; TRM: resident memory T cell). (B) Dot plot visualization of gene markers used for cell identification of myeloid cells in dura and tumor samples.


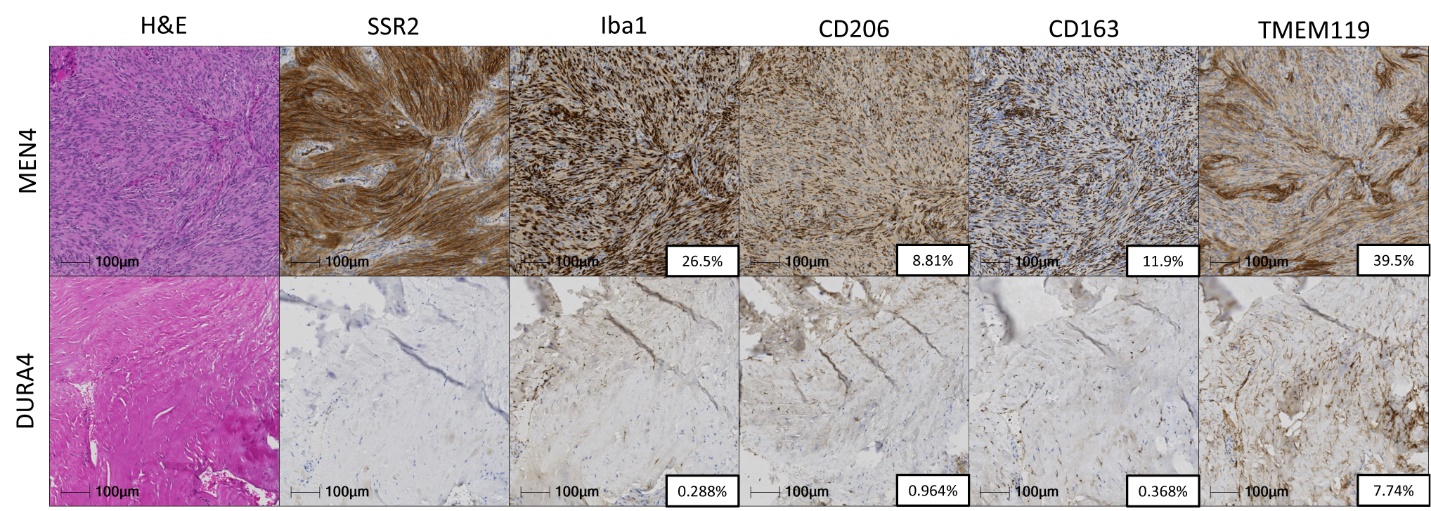


**Fig. S5. Immunohistochemistry of a matched pair of non-tumor associated dura and tumor.** Immunohistochemical staining of somatostatin receptor 2 (SSR2), Iba1, CD206, CD163, and TMEM119 in a matched pair of non-tumor associated dura and meningioma tumor (SAMPLE104). Insets contain %-positive area for selected marker.


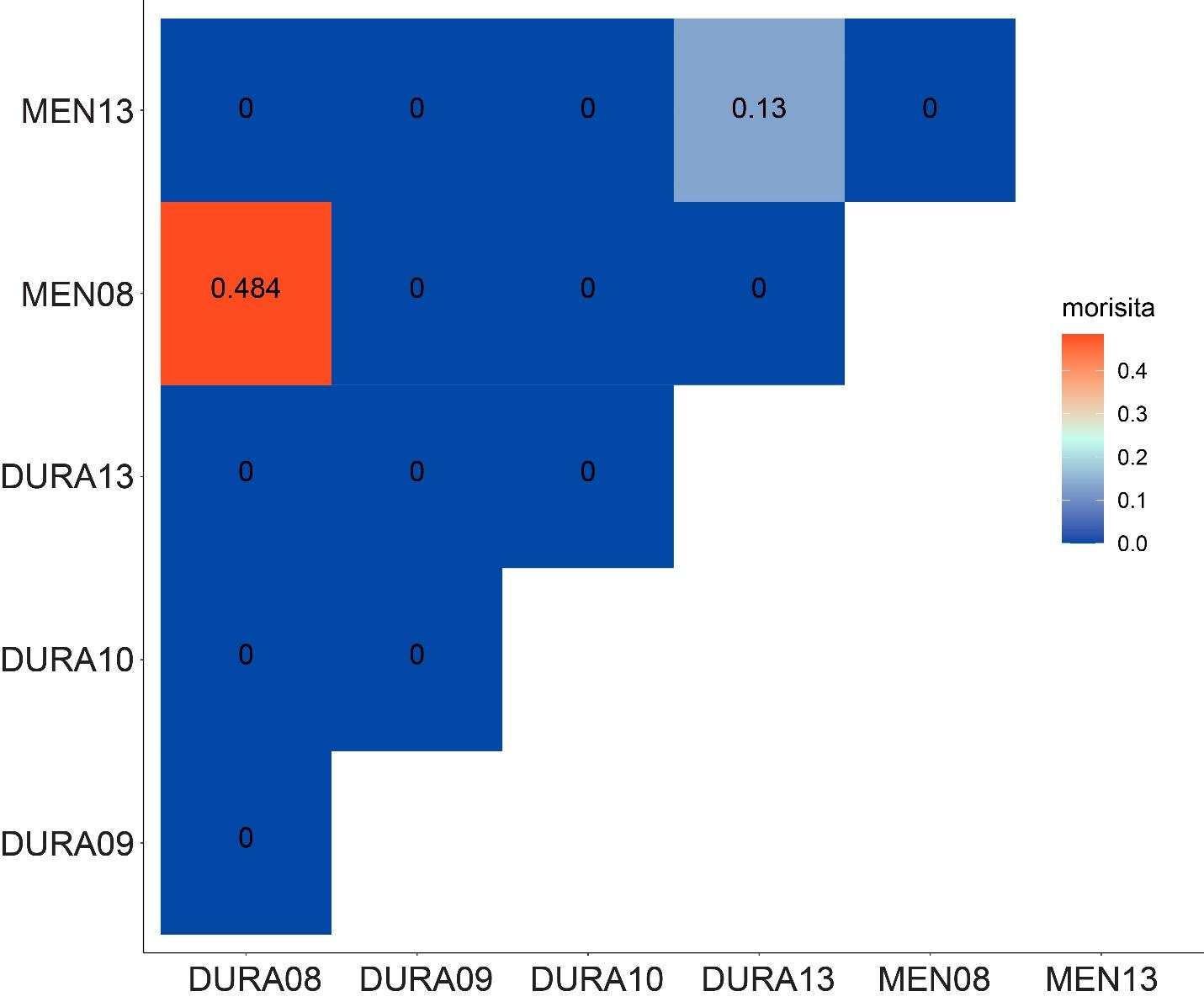


**Fig. S6. Quantification of TCR overlap.** The Morisita overlap index was calculated comparing the overall TCR repertoires from respective dura and tumor samples. Values range from 0 (no similarity) to 1 (completely identical TCR repertoires).


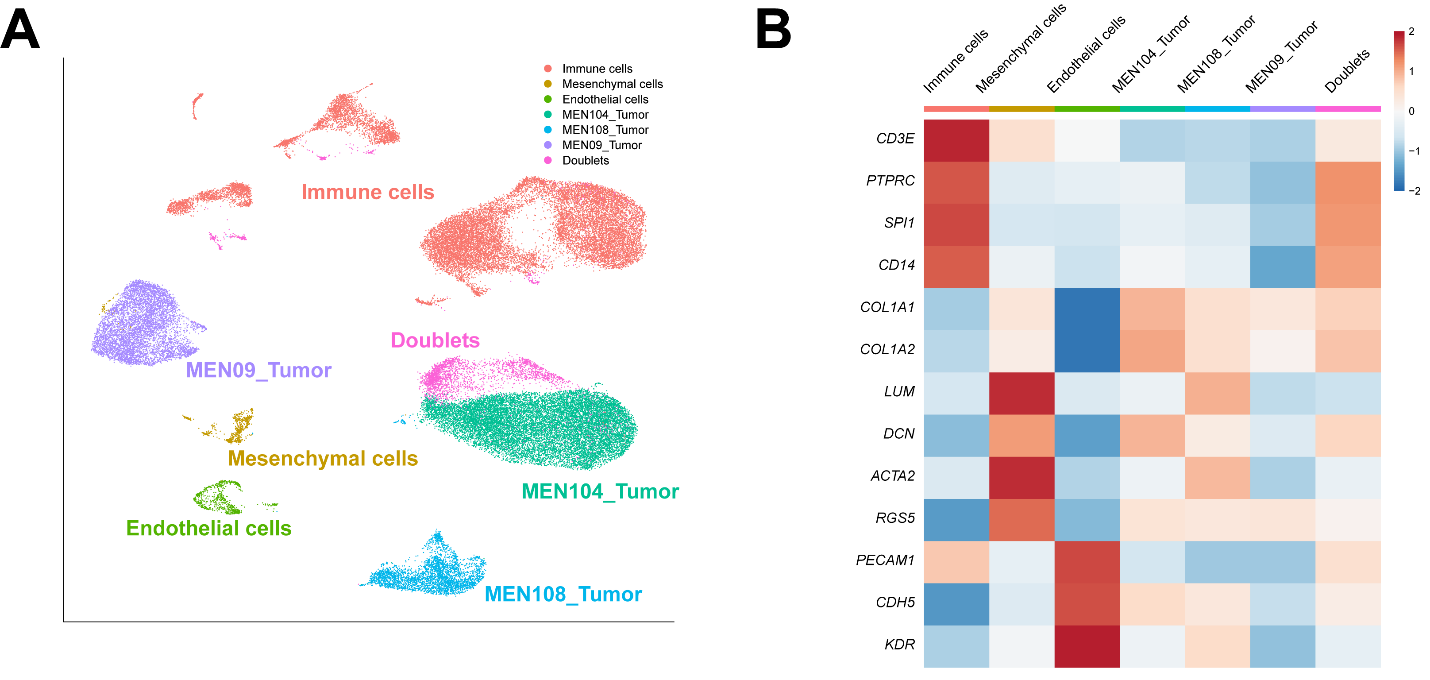


**Fig. S7. Analysis of DURA09, MEN09, MEN104, and MEN108 samples differentiated by general cell types. (A)** UMAP visualization of general cell types in DURA09, MEN09, MEN104, AND MEN108. **(B)** General markers used to differentiate specific cell types in Fig. S7A.


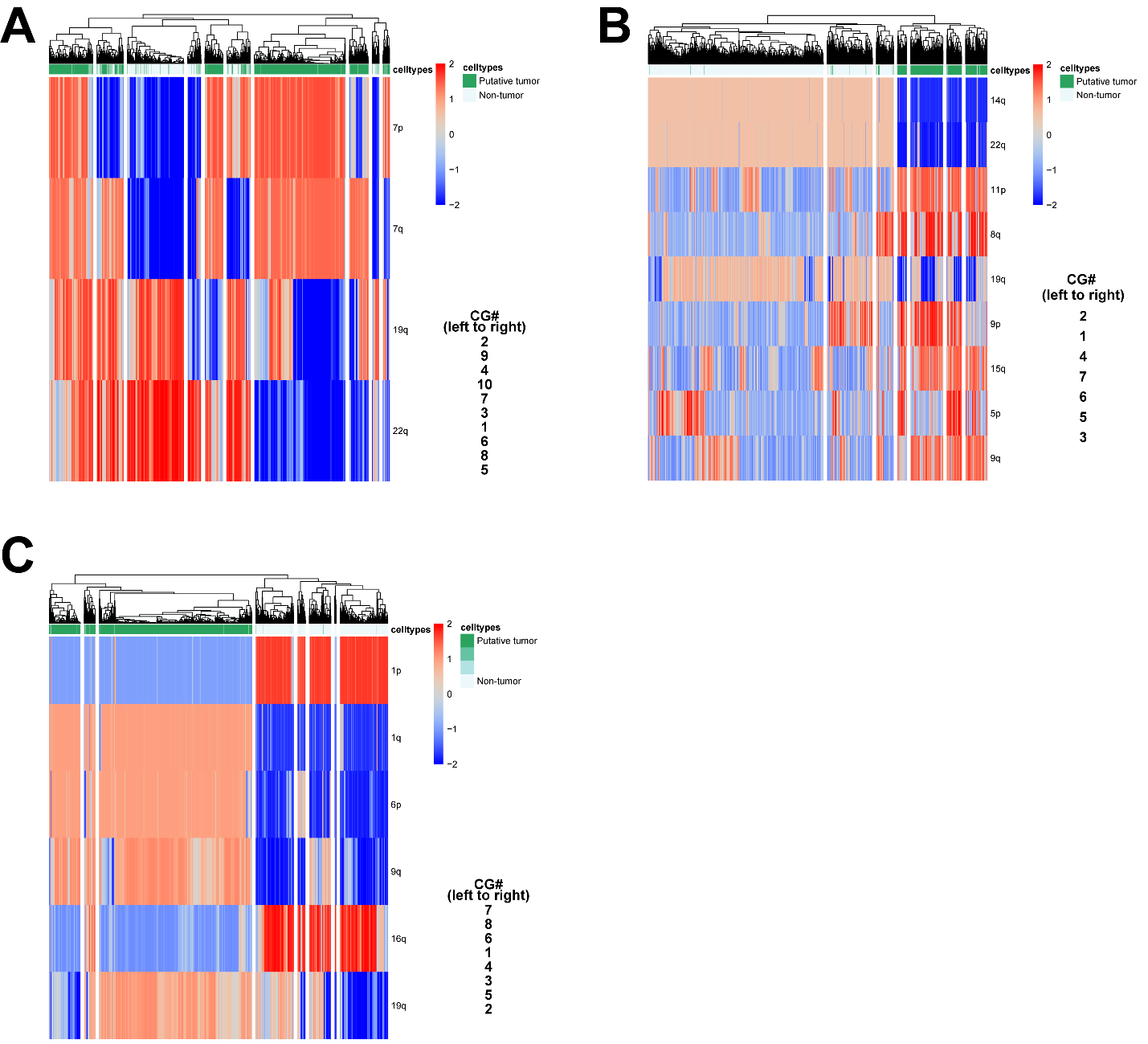


**Fig. S8. CONICSmat analysis of putative patient specific tumor cells. (A-C)** CONICSmat output of CNV analysis of patient specific tumor cells. Putative tumor cells were compared to patient matched non-tumor cells (*PTPRC*+ cells) to estimate CNVs. **A:** MEN104; **B:** MEN105; **C:** MEN09.

**Table S1. Patient demographics and sample characteristics.** Basic demographic information and sample characteristics.

| **Sample Names** | **Patient Gender** | **Tumor Location** | **Pathology** | **Tissue Type** | **Analyses performed** |
| --- | --- | --- | --- | --- | --- |
| **SAMPLE02** | **F** | **L sphenoid wing** | **WHO grade II, atypical** | **Non-tumor associated dura (DURA02)** | **IMC** |
| **SAMPLE05** | **M** | **R sphenoid wing** | **WHO grade II, atypical** | **Non-tumor associated dura (DURA05)** | **3’ scRNAseq, IMC** |
| **SAMPLE06*** | **F** | **R frontotemporal** | **WHO grade II, atypical** | **Non-tumor associated dura (DURA06)** | **3’ scRNAseq** |
| **SAMPLE08** | **M** | **L frontal** | **WHO grade I, angiomatous** | **Non-tumor associated dura (DURA08)** | **5’ scRNAseq, VDJ enrichment** |
|  |  |  |  | **Meningioma (MEN08)** | **5’ scRNAseq, VDJ enrichment** |
| **SAMPLE09** | **F** | **L frontal** | **WHO grade III, anaplastic** | **Non-tumor associated dura (DURA09)** | **5’ scRNAseq, VDJ enrichment** |
|  |  |  |  | **Meningioma (MEN09)** | **5’ scRNAseq** |
| **SAMPLE10** | **M** | **L frontal** | **WHO grade I** | **Non-tumor associated dura (DURA10)** | **5’ scRNAseq, VDJ enrichment** |
|  |  |  |  | **Meningioma (MEN10)** | **5’ scRNAseq** |
| **SAMPLE11** | **M** | **Parafalcine** | **WHO grade II, atypical with patchy angiomatous** | **Non-tumor associated dura (DURA11)** | **5’ scRNAseq** |
| **SAMPLE13** | **F** | **Parafalcine** | **WHO grade II, atypical** | **Non-tumor associated dura (DURA13)** | **5’ scRNAseq, VDJ enrichment** |
|  |  |  |  | **Meningioma (MEN13)** | **5’ scRNAseq, VDJ enrichment** |
| **SAMPLE104** | **F** | **R temperoparietal** | **WHO grade I** | **Meningioma**  **(MEN104.1- CD45pos fraction, MEN104.2-CD45neg fraction)** | **5’ scRNAseq, IHC** |
| **SAMPLE108** | **M** | **R parasagittal** | **WHO grade II, atypical** | **Meningioma**  **(MEN108.1- CD45pos fraction, MEN108.2-CD45neg fraction)** | **5’ scRNAseq** |

*Collected from a patient undergoing surgical resection for a recurrent meningioma. All other samples are collected from patients undergoing surgical resection for a primary meningioma.

**Table S2. Gene markers for cell type identification.** Markers used to characterize cell identity of obtained clusters based upon current literature.

| Cell type | Markers | Ref. |
| --- | --- | --- |
| Endothelial cell | *PECAM1, CDH5, KDR, SELE, VWF* | 40, 41 |
| Fenestrated endothelium | *PLVAP, PLPP1, PLPP3, NRP1, ESM1, RGCC* | 51 |
| Blood-brain barrier endothelium | *FZD1-10, ADGRA2, CTNNB1, CDH5, SLC2A1, TNFRSF21, TNFRSF19, CLDN1, CLDN3, CLDN5, CLDN12, LSR, OCLN, TJP1, TJP2, TJP3, SLC5A1, SLCO1A2, SLC22A8, SLC16A1, PGP, ABCG2, ABCC4* | 50 |
| Mesenchymal cell | *COL1A1, COL1A2, LUM, DCN, ACTA2, RGS5* |  |
| Immune cell | *PTPRC, CD3E, SPI1, CD14* |  |
| Naïve/central memory T cell (TCM) | *SELL, CCR7, LEF1, TCF7, KLF2* | 29 |
| CD4+ effector memory T cell (TEM) | *SELL-, CCR7-, IL7R* | 30 |
| CD8+ TEM | *SELL-, CCR7-, IL7R, CD8A, CD8B* | 30 |
| Resident memory T cell (TRM) | *CD69, NR4A2, IL7R* | 30 |
| CD8+ cytotoxic T cell (CTL) | *PRF1, NKG7, ZNF683, GZMB, CD8A, CD8B* | 29 |
| Natural killer cell | *PRF1, NKG7, GZMB, KLRD1, KLRF1, CD3-* | 31 |
| B cell | *CD79A, MS4A1,* MHC class II | 32 |
| Plasma-like B cells | *IGHG3, IGHA1, DERL1, FKBP11* | 32 |
| Monocyte/macrophage | *CD14, VCAN, S100A8, S100A9, RNASE1, C1QA, C1QC, FCAR, GPNMB,* MHC class II | 33-36 |
| Microglia | *AIF1, GPR34, CD14, C1QA, C1QB, C1QC* | 26 |
| Border associated macrophage (BAM) | *MRC1, STAB1, CD163, APOE, MS4A7* | 10 |
| MHCII^lo^ MDSC-like | Monocyte/macrophage genes, MHC class II- | 38 |
| DC-like | *CD14-, ITGAX, THBD,* MHC class II | 33, 35 |
| migDC-like | *CD14-, IL3RA, CCR7, IDO1, LAD1, CCL22, RAMP1,* MHC class II | 33, 35, 36 |
| Mast cell | *GATA2, KIT, HPGDS* | 37 |
| Fibroblast | *LUM, DCN, COL1A1, COL1A2, COL3A1* | 40, 41 |
| Mural cell | *ACTA2, MYH11, CNN1, RGS5, PDGFRB, NOTCH3, MCAM, CSPG4* | 41-43 |

**Table S3. Antibody Information.** Antibody targets with respective conjugated heavy metals (if used for IMC), dilution factor, antibody clone, clone number, and manufacturer information. All conjugated antibodies were at a concentration of 0.5 mg/mL except for Iba1 (0.336 mg/mL). Otherwise, antibodies were at stock concentration.

| **Target** | **Label** | **Dilution factor** | **Clone** | **Manufacturer** | **Catalog #** |
| --- | --- | --- | --- | --- | --- |
| **a-SMA** | **089Y** | **1:50** | **1A4** | **eBiosciences** | **14-9760-82** |
| **CD14** | **144Nd** | **1:400** | **3144025D** | **Fluidigm** | **EPR3653** |
| **CD163** | **147Sm** | **1:200** | **EDHu-1** | **Fluidigm** | **3147021D** |
| **CD11b** | **149Sm** | **1:100** | **EPR1344** | **Fluidigm** | **3149028D** |
| **CD31** | **151Eu** | **1:75** | **EPR3094** | **Fluidigm** | **3151025D** |
| **CD4** | **156Gd** | **1:450** | **EPR6855** | **Fluidigm** | **3156033D** |
| **Iba1** | **158Gd** | **1:500** | **Poly** | **Novusbio** | **NBP2-19019** |
| **CD8a** | **162Dy** | **1:375** | **C8/144B** | **Fluidigm** | **3162034D** |
| **CD45RA** | **166Er** | **1:1000** | **PTPRC/818** | **Novusbio** | **NBP2-47957** |
| **GZMB** | **167Er** | **1:500** | **EPR20129-217** | **Fluidigm** | **3167021D** |
| **Collagen I** | **169Tm** | **1:2500** | **Polyclonal** | **Fluidigm** | **3169023D** |
| **CD3** | **170Er** | **1:100** | **polyclonal C-Terminal** | **Fluidigm** | **3170019D** |
| **CD45RO** | **173Yb** | **1:300** | **UCHL1** | **Fluidigm** | **3173016D** |
| **HLA-DR** | **174Yb** | **1:350** | **LN3** | **Fluidigm** | **3174025D** |
| **Histone H3** | **176Yb** | **1:2000** | **D1H2** | **Fluidigm** | **3176023D** |
| **DNA-Ir1** | **191Ir** | **1:400** | **125uM** | **Fluidigm** | **201192A** |
| **DNA-Ir2** | **193Ir** | **1:400** | **125uM** | **Fluidigm** | **201192A** |
| **CD206** | **IHC** | **1:2000** |  | **abcam** | **ab64693** |
| **CD163** | **IHC** | **1:200** |  | **Leica** | **NCL-L-CD163** |
| **Iba1** | **IHC** | **1:200** | **EPR16588** | **abcam** | **ab178846** |
| **TMEM119** | **IHC** | **1:250** | **C-terminal** | **abcam** | **ab185333** |
| **Somatostatin receptor 2** | **IHC** | **1:1000** | **UMB1-C-terminal** | **abcam** | **ab134152** |

**Table S4. Gene markers for macrophage polarization states.** Markers associated with macrophage polarization states.

| Polarization state | Markers | Ref. |
| --- | --- | --- |
| M1 macrophage polarization | *CD86, CD80, IL1R1, TLR2, TLR4, NOS2, SOCS3, IL1B, TNF, IL6, FCGR2A, MARCO, NOS2, NFKB1, STAT1, IRF5, JUN, FCGR1A, IDO1, SOCS1, CXCL10* | 25 |
| M2a macrophage polarization | *CD163, MRC1, TGFB1, SLAMF1, SPHK1, THBS1, HMOX1, STATE3, TLR1, TLR8, TGM2, FCER2, CCL22* | 25 |
| M2b macrophage polarization | *MRC1, TREM2, IGF1, IL1RN, STAT6, KLF2, IRF4, PPARG, PPARD, CD163, TGM2, IL1R2, FCER2, CCL22* | 25 |
| M2c macrophage polarization | *IL6, VEGFA, IGF1, CD86, TNF, FCGR1A, MRC1, TGM2, FCER2, CCL22* | 25 |
